# Near-infrared luminescent open-shell π-conjugated systems with a bright lowest-energy zwitterionic singlet excited state

**DOI:** 10.1126/sciadv.ado3476

**Published:** 2024-07-24

**Authors:** Craig P. Yu, Rituparno Chowdhury, Yao Fu, Pratyush Ghosh, Weixuan Zeng, Tarig B. E. Mustafa, Jeannine Grüne, Lucy E. Walker, Daniel G. Congrave, Xian Wei Chua, Petri Murto, Akshay Rao, Henning Sirringhaus, Felix Plasser, Clare P. Grey, Richard H. Friend, Hugo Bronstein

**Affiliations:** ^1^ Yusuf Hamied Department of Chemistry, University of Cambridge, Cambridge CB2 1EW, UK.; ^2^ Department of Physics, Cavendish Laboratory, Cambridge University, Cambridge CB3 0HF, UK.; ^3^ Department of Chemistry, Loughborough University, Loughborough, LE11 3TU, UK.

## Abstract

Open-shell systems with extensive π-conjugation have fascinating properties due to their narrow bandgaps and spin interactions. In this work, we report neutral open-shell di- and polyradical conjugated materials exhibiting intriguing optical and magnetic properties. Our key design advance is the planarized geometry allowing for greater interaction between adjacent spins. This results in absorption and emission in the near infrared at 803 and 1050 nanometers, respectively, and we demonstrate a unique electronic structure where a bright zwitterionic excited state is the lowest-accessible electronic transition. Electron paramagnetic resonance spectroscopy and superconducting quantum interference device measurements reveal that our materials are open-shell singlets with different degrees of spin interactions, dynamics, and antiferromagnetic properties, which likely contributed to the formation of their emissive zwitterionic singlet excited state and near-infrared emission. In addition, our materials show reversible and stable electrochromic switching with more than 500 cycles, indicating their potential for optoelectronic and electrochemical energy storage applications.

## Teaser

Open-shell systems exhibit near-infrared absorption and emission via a bright lowest-energy zwitterionic singlet excited state.

## INTRODUCTION

Neutral open-shell π-conjugated molecules demonstrate fascinating optoelectronic and magnetic properties (*1*–*3*). Key advances in synthetic chemistry not only allowed more in-depth understanding of open-shell π-conjugated systems, but they also led to the development of radical-based electronics with applications (*4*–*6*) in organic field-effect transistors (*7*) and spintronics (*8*). In contrast to the conventional closed-shell optoelectronic materials, which always have lower-energy dark triplet states (*9*), open-shell emitters have spin-allowed relaxation from the doublet excited state (D_1_) to the doublet ground state (D_0_) as the lowest-energy optical transition (*10*, *11*). However, almost all open-shell systems are poorly luminescent due to a combination of their usual weakly forbidden optical transitions and the energy gap law. Symmetric tris(2,4,6-trichlorophenyl)methyl (TTM) systems, where the radical is highly stabilized by the *o*-chlorine atoms via steric and electronic effects, are typically poorly emissive due to its symmetry-induced cancellation of its transition dipole moment. However, upon functionalization of TTM with electron-rich moieties, the oscillator strength of its lowest-energy transition is increased by approximately an order of magnitude. Consequently, these materials can show outstanding luminescence properties with photoluminescence quantum efficiencies (PLQEs) approaching 100% and excellent external quantum efficiencies in OLEDs (*1*, *11*, *12*). Di- and polyradical conjugated systems have the additional possibility of interactions between the unpaired electrons leading to open-shell singlet, triplet, and higher multiplicities in the ground and excited states which may also lead to intriguing magneto and spin-active properties (*13*). These materials are typically made from either linking together open-shell mono-radical containing small molecules or by combining very strong donors and acceptors to achieve narrow bandgaps such that open-shell electronic configurations are thermodynamically favorable. However, these materials have very rarely been shown to be emissive and are often very unstable under ambient conditions. Thus, the design and synthesis of stable open-shell polyradical systems with strong absorptive and emissive properties are essentially unknown.

We recently showed mesitylation of TTM in the *para* position (M*_n_*TTM) to be a simple yet powerful strategy to provide synthetic versatility, stability, and enhanced photoluminescence in these materials (*14*). The fluorene-M_1_TTM polymer (PFMTTM) polyradical exhibited a deep-red, near-infrared (NIR) emission at 805 nm, with a PLQE of 15%. Motivated by this, we sought to explore the effect of conjugation and delocalization on the optical and spin properties of conjugated polyradicals. The current design of TTM-based emitters has been largely focused on introducing moieties such as carbazole and fluorene to improve the charge transfer character of the doublet exciton. Enhancing the conjugation between TTM radical centers by introducing highly planar π-linkers has not been experimentally investigated. Achieving NIR emission in closed-shell organic semiconductors has been a great challenge due to the energy gap law and their low-energy dark triplet states (*15*), and a highly planar and π-conjugated open-shell system based on the M*_n_*TTM design is expected to exhibit emission even more into the NIR region, offering a new route towards addressing this challenge. In addition, by improving the π-conjugation between TTM radical centers within an open-shell system, correlation between the unpaired electrons may be expected, allowing us to probe the intrinsic spin-spin interactions and dynamics that can pave the path for magneto-optic and spintronic applications.

**Fig. 1.**
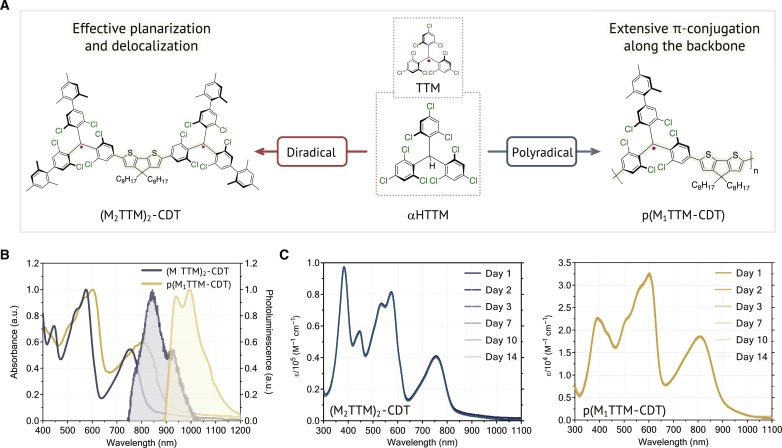
Structures and photophysical properties of (M_2_TTM)_2_-CDT and p(M_1_TTM-CDT). (**A**) Design and molecular structures of planarized di- and polyradicals. M, mesityl group. (**B**) Solution-state absorption and emission of (M_2_TTM)_2_-CDT and p(M_1_TTM-CDT) and their (**C**) time-dependent UV-vis absorption spectra in toluene with a concentration of 5.0 × 10^−5^ M. a.u., arbitrary units.

Here, we present an effective design using cyclopenta[2,1-*b*:3,4-*b′*]dithiophene (CDT) (*16*) as a π-linker that connects the M*_n_*TTM unit in diradical (M_2_TTM)_2_-CDT and polyradical p(M_1_TTM-CDT) open-shell systems, where the radicals are able to interact to a greater extent than the existing TTM systems (Fig. 1A). The extensive π-conjugation that the CDT moiety provides to the entire system contributes to a highly planarized and delocalized molecular orbital and spin density distributions along the backbone (Fig. 1A and fig. S1A), allowing for exciton delocalization and spin-spin interactions. (M_2_TTM)_2_-CDT and p(M_1_TTM-CDT) show intense NIR absorption bands that are due to an allowed optical transition to a zwitterionic singlet excited state as the lowest-energy excited state, and this absorption feature is completely absent in the control monoradical M_2_TTM-CDT-Ph material. Formation of the zwitterionic excited states in (M_2_TTM)_2_-CDT and p(M_1_TTM-CDT) strongly suggests effective delocalization of the unpaired spins supported by π-conjugation along the backbones. Temperature-dependent electron paramagnetic resonance (EPR) and superconducting quantum interference device (SQUID) measurements indicate that the current systems exhibit delocalized and antiferromagnetic coupling between the unpaired spins. Last, we demonstrate reversible electrochromic switching in the NIR-I and NIR-II regions that could be promising for radiative cooling (*17*), showing that conjugated polyradicals are a powerful and important class of materials for next-generation spin- and optoelectronic applications.

## RESULTS

### Synthesis of open-shell systems

To synthesize the (M_2_TTM)_2_-CDT diradical (fig. S1B), we started from the previously reported aHM_2_TTM-Bpin and performed a Suzuki-Miyaura cross coupling with 2,6-dibromo-4,4-dioctyl-4*H*-cyclopenta[2,1-*b*:3,4-*b′*]dithiophene using Pd(OAc)_2_/SPhos as the catalyst. The product (aHM_2_TTM)_2_-CDT was obtained in 78% yield after 24 hours of heating at 80°C. Similarly, the polymer precursor, p(aHM_1_TTM-CDT) was synthesized from a Suzuki-Miyaura polycondensation between aHM_1_TTM-Bpin_2_ and 2,6-dibromo-4,4-dioctyl-4*H*-cyclopenta[2,1-*b*:3,4-*b′*]dithiophene under microwave irradiation, giving the target polymer in 81% yield. The alpha hydrogen of (aHM_2_TTM)_2_-CDT is deprotonated using *tert*-butylammonium hydroxide in a mixture of tetrahydrofuran and dimethyl sulfoxide to give the anionic intermediate, where the reaction kinetics were predetermined by ^1^H nuclear magnetic resonance (NMR), and the neutral diradical (M_2_TTM)_2_-CDT was generated by the addition of *p*-chloranil. The deprotonation of p(aHM_1_TTM-CDT) was monitored by ultraviolet-visible (UV-vis) by observing total bleaching of the polymer precursor absorption (fig. S2), and the subsequent addition of *p*-chloranil oxidized the polyanionic intermediate to furnish the target p(M_1_TTM-CDT) polyradical with a number averaged molecular weight of 8.6 kg/mol and a dispersity of 1.45. (M_2_TTM)_2_-CDT and p(M_1_TTM-CDT) appear to be thermally stable above 300°C (fig. S3).

### Photophysical properties

(M_2_TTM)_2_-CDT and p(M_1_TTM-CDT) both exhibit broad UV-vis absorption features in solution and thin films, with intense absorption bands at 756 and 803 nm, respectively (Fig. 1B). Various high-spin π-conjugated systems have displayed absorptions in the NIR region due to the narrow highest occupied molecular orbital–singly occupied molecular orbital (HOMO-SOMO) energy gap (*5*), but broad absorptions with such large extinction coefficients for the lowest-energy transitions are rarely observed. The lowest-energy bands of (M_2_TTM)_2_-CDT and p(M_1_TTM-CDT) are more than one order of magnitude higher than that of the reported TTM-3PCz (*18*), suggesting that the enhancement of π-conjugation between radical centers may be effective in increasing the optical absorptions. The (M_2_TTM)_2_-CDT diradical exhibits higher molar extinction coefficients than previously reported diradical structures (*7*, *19*, *20*), p(M_1_TTM-CDT) also shows comparable or higher molar extinction coefficients than other polyradicals (*21*, *22*), as well as regular closed-shell π-conjugated polymers (*23*). Both materials are very stable at ambient conditions with no observable photodegradation over the course of 14 days (solutions were stored in sealed vials in dark) (Fig. 1C). Although TTM-based emitters frequently demonstrate deep-red photoluminescence, their emission wavelengths have rarely been reported to be above 800 nm (*24*–*27*). In addition, di- and polyradical organic π-conjugated systems generally show completely quenched fluorescence in this spectral region (*28*–*30*). Intriguingly, the diradical with the CDT π-linker shows a photoluminescence λ_PL_ of 870 nm with a 0.8% PLQE in toluene solution (Fig. 1B). The polyradical p(M_1_TTM-CDT), on the other hand, demonstrates an even more red-shifted solution-state emission at λ_PL_ of 1050 nm with a PLQE of 0.2%, which is well into the NIR-II region (Fig. 1B). In neat films, (M_2_TTM)_2_-CDT and p(M_1_TTM-CDT) retain NIR emissions at 853 and 945 nm albeit with low PLQE (<0.1%) due to solid-state quenching effects (fig. S4A). While the PLQEs of current open-shell materials may appear modest, their emissions at these NIR wavelengths are indeed remarkable, and these results place our materials among the small class of NIR-emitting organic polymers with emissions beyond 1000 nm (*31*–*33*). We performed femtosecond transient absorption spectroscopy (fig. S5A) to understand the nature of the exciton and found that the excited state displays a notable biexponential behavior with initial decay lifetimes of 5 and 8 ps in di- and polyradicals, respectively (fig. S5B). This fast initial drop suggests strong exciton-phonon coupling providing facile nonradiative channels. Following this initial decay, about 1% of the exciton remains in the relaxed excited state which provides the population for the radiative decay that decays within 1 ns.

### The zwitterionic singlet excited state

**Fig. 2.**
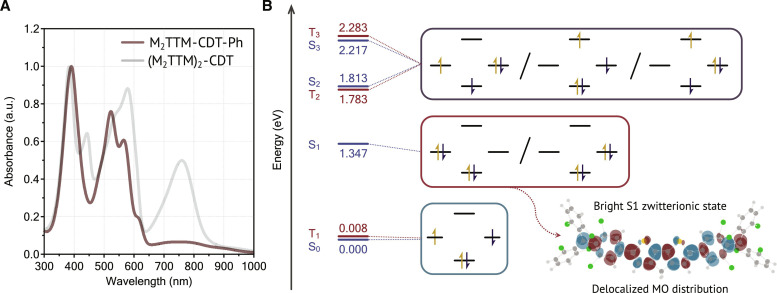
Evidence of the zwitterionic excited state. (**A**) Normalized solution-state UV-vis absorption spectra of mono- and diradicals in toluene. (**B**) Lowest excited singlet (blue) and triplet (red) states with vertical excitation energies in electron volts, dominant configurations, and transition density of the bright S1 state.

The unusually intense absorption bands in the NIR region and photoluminescence of (M_2_TTM)_2_-CDT and p(M_1_TTM-CDT) prompted us to further investigate their electronic structures. After synthesizing the monoradical analog, M_2_TTM-CDT-Ph (fig. S1B), we noticed that the strong long-wavelength absorption is completely absent (Fig. 2A), which strongly suggests that this feature is attributed to the effect of more than one unpaired spin in the π-conjugated system. To elucidate the electronic structure of these molecules in more detail, we performed computations using high-level ab initio multireference methods [2nd order complete active space perturbation theory (CASPT2)] (*34*). Computations were performed on (M_2_TTM)_2_-CDT with the outer mesityl rings removed, and the low-lying singlet and triplet states were computed. The frontier orbitals (computed as state-averaged natural orbitals) of this molecule are presented in fig. S6, showing that the HOMO and lowest unoccupied molecular orbital (LUMO) are not only predominantly localized on the central CDT group but also slightly delocalized onto the TTM groups. The SOMOs are largely focused on the central carbon atoms of TTM units with notable delocalization along the molecule as well. Note that we have localized the SOMOs on either left or right of the CDT group, respectively, to facilitate the subsequent analysis in terms of ionic and radical characters.

The lowest singlet and triplet excited states are presented in Fig. 2B, with singlets shown in blue and triplets in red. The respective configurations, following the orbitals presented in fig. S6, are shown as insets (full information in tables S1 and S2 and figs. S7 and S8). In the case of weakly interacting radical centers, one would expect the states to come in quasi-degenerate singlet/triplet pairs with analogous electronic configurations, each derived from one of the monomer doublet states. The notable observation is that this is true for all states except for one, and this exception is the bright zwitterionic state at 1.347 eV responsible for the unique NIR optical properties of this material. Starting the discussion with the two lowest states (S_0_ and T_1_), we find that both are strongly dominated by a diradical SOMO^2^ configuration (fig. S6). These states are almost degenerate with T_1_ lying slightly higher than S_0_ (by 8 meV), which is in good agreement with magnetic measurements (see below). Next comes the bright S_1_ state lying at 1.347 eV and having an oscillator strength of 0.275. The two dominant configurations, when expressed within a basis of localized SOMOs, are shown on the right. These configurations are characterized by the fact that both electrons are either simultaneously on the left or the right radical center, and the overall state is an even mixture of both configurations. States of this type are termed zwitterionic (or simply ionic) (*35*–*37*). The decisive feature of the zwitterionic state is that no analog exists in the monoradical M_2_TTM-CDT-Ph, since the zwitterionic state is derived from charged monomer states and that no analog exists in the triplet manifold due to the Pauli principle. These two properties explain the unique features of the zwitterionic state: Why it emerges only in the di- and polyradicals, and why no triplet exists at a similar energy level. We also want to point out that the ionic SOMO^2^ configuration accounts for only about 50% of the overall wave function character. Another 25% are given by a HOMO → SOMO excitation strongly involving the bridging CDT unit. This finding shows that not only the zwitterionic character of the S_1_ determines its optical properties, but it is also decisively affected by the delocalization onto the bridging CDT unit. To illustrate the delocalization, we plot the transition density (Fig. 2B). This shows that the transition density is delocalized from the left to the right radical centers with notable contributions on the whole bridge in between. Moving further up in energy, we find that S_2_, T_2_, S_3_, and T_3_ are all predominantly composed of HOMO → SOMO, SOMO → LUMO, and HOMO → LUMO configurations, respectively, in various mixtures. These, again, are states where clear analogs exist in the monoradical. We note that no second zwitterionic state, as expected for an idealized diradical (*36*), is found in the energy range studied here, again highlighting the strong involvement of the bridging CDT unit. The (M_2_TTM)_2_-CDT diradical exhibits solvatochromism and solvatofluorochromism, where pronounced red shifts are observed with increased solvent polarity (fig. S9), and the solvent effect on the optical absorption of (M_2_TTM)_2_-CDT is also supported by our calculations (table S3). This demonstrates the polar nature of the zwitterionic excited state and the pronounced solvatochromism in the optical absorption further substantiates the zwitterionic excited state being the lowest-accessible excited state.

To summarize, the unique optical properties of (M_2_TTM)_2_-CDT are determined by the emergence of a zwitterionic singlet state with no analog in the monoradical and no analogous state in the triplet manifold. Mixing with the HOMO → SOMO transition renders this into a delocalized state lying at low energy and having a high oscillator strength. An extrapolation to the p(M_1_TTM-CDT) polyradical can be done in the following way. The ground state of the polyradical is expected to be an open-shell singlet state with antiferromagnetically coupled electrons residing on the individual radical centers. A state, analogous to the S_1_ of (M_2_TTM)_2_-CDT, can now be formed by displacing one of the unpaired electrons and moving it to an adjacent site. Whereas in (M_2_TTM)_2_-CDT, this process leads to one low-energy excited state; in p(M_1_TTM-CDT), one can construct a band of delocalized excitonically coupled states of related character. However, aside from excitonic splitting, it is reasonable to assume that the state in the polyradical will behave analogously to the diradical. Zwitterionic states in diradicals have been previously reported (*38*–*40*), but a key difference is that our systems represent the first example where this state is the lowest-accessible excited state in both absorption and emission. The consequence of this is that our materials are able to both strongly absorb and emit NIR light (comparably if not better than conventional closed-shell conjugated polymers) but do not have any lower-lying dark excited states, suggesting that these may be of use in OLED and OPV applications where nonradiative recombination (often to lower-lying triplets) is the major source of efficiency loss in current materials.

### Understanding the spin-spin interactions

**Fig. 3.**
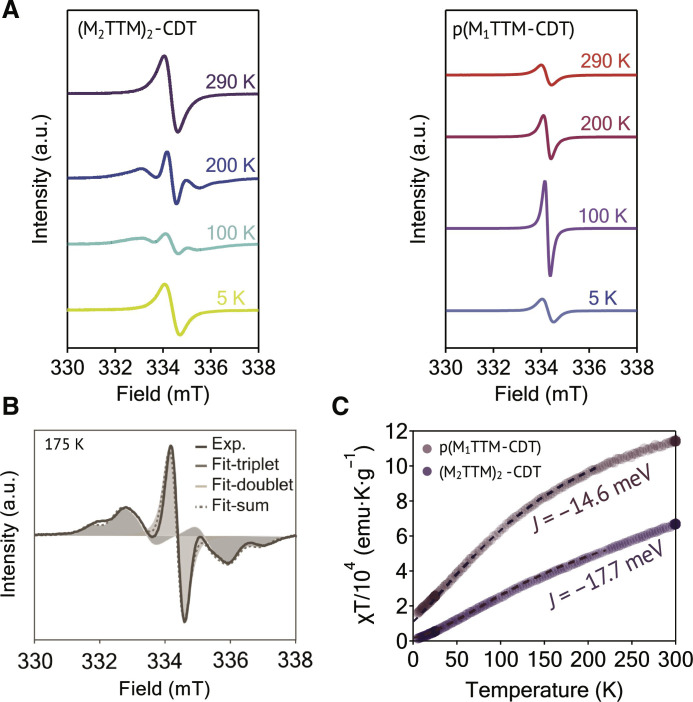
Investigation of the spin properties of (M_2_TTM)_2_-CDT and p(M_1_TTM-CDT). (**A**) Temperature-variant EPR spectra in diluted toluene solutions with a concentration of 50 μm. (**B**) The EPR fitting plot of (M_2_TTM)_2_-CDT indicating the simulated doublet and triplet spectra and the experimental spectrum at 175 K. (**C**) χT-T plot of (M_2_TTM)_2_-CDT and p(M_1_TTM-CDT) powdered samples. The dotted fitting curves are obtained using the Bleaney-Bowers equation.

To understand the interactions between the unpaired spins, we acquired continuous-wave EPR spectra of (M_2_TTM)_2_-CDT and p(M_1_TTM-CDT) in toluene solutions (50 μM) from 290 to 5 K. Both samples exhibit a similar *g* value from 2.0026 to 2.0029 across the entire temperature range (tables S4 and S5). In the (M_2_TTM)_2_-CDT solution, a spin-spin interaction (*41*) emerges at 230 K (Fig. 3A and fig. S10), indicated by the zero-field splitting (ZFS) broadened signal arising from the formation of a triplet state (Fig. 3B). The (M_2_TTM)_2_-CDT diluted film [1 wt % in a poly(methyl methacrylate) matrix; fig. S12], on the other hand, shows ZFS starting from 290 K, suggesting that the apparent absence of ZFS from 290 to 230 K in its solution state is the result of thermally coupled structural dynamics. This spin-spin interaction (dipolar interaction) is most likely enabled by the enhanced planarity and conjugation between the unpaired spins. In both solution and film states, the spin-spin distance estimated by a point-dipole approximation method (tables S4 and S6) (*42*) is substantially shorter than the density functional theory calculated distance (*43*) between two radical centers (1.77 nm), suggesting delocalized triplet states (*44*). However, the ZFS splitting pattern resulting from dipolar interactions is no longer visible as the temperature drops below 10 K, which might be attributed to a structural change to a nonplanar geometry that disallows the spin correlations.

A phenyl-bridged TTM diradical system (TTM-phTTM) with a thermally accessible triplet state was recently reported (*45*). Despite the two spatially close radical centers in this system, no spectral signatures of dipolar interactions were seen in its temperature-dependent EPR spectra, which further supports the important role played by the introduction of a planar π-conjugation unit for promoting spin-spin interactions. The polyradical p(M_1_TTM-CDT), on the other hand, does not show the ZFS pattern or dipolar interactions observed in (M_2_TTM)_2_-CDT (Fig. 3A and fig. S11); this may be attributed to the more disordered or dynamic nature of the polyradical chains, leading to no noticeable spin-spin interactions (*46*, *47*).

Current open-shell systems were investigated using SQUID to elucidate their magnetic properties. Both materials demonstrate a negative temperature dependence of χT (Fig. 3C), suggesting antiferromagnetic exchanges between unpaired spins (*48*). By fitting the χT curves using the Bleaney-Bowers equation, *J* values of −17.7 and −14.6 meV for (M_2_TTM)_2_-CDT and p(M_1_TTM-CDT), respectively, were obtained, which indicates their singlet-triplet energy gaps, and the negative *J* values suggest an open-shell singlet ground state for these two materials. Temperature-dependent effective magnetic moments (μ_eff_) were also extracted, (M_2_TTM)_2_-CDT showing a gradual decrease of μ_eff_ from 3.03 μ_B_ at 300 K to 0.457 μ_B_ at 5.69 K. Similarly, the μ_eff_ of p(M_1_TTM-CDT) decreased from 300 K at 8.85 to 3.29 μ_B_ when temperature was lowered to 5 K. Both μ_eff_ values at 300 K correlate well with the expected number of unpaired spins [eight for p(M_1_TTM-CDT) and two for (M_2_TTM)_2_-CDT; fig. S13], and the negative temperature dependence of μ_eff_ further suggests antiferromagnetic interactions in their ground states. The collective EPR and SQUID data herein demonstrate that the CDT-containing open-shell systems with extensive π-conjugations have controllable spin-spin interactions by tuning their repeat units. The open-shell singlet ground state with antiferromagnetic exchange may lead to fascinating spintronic applications (*49*, *50*).

### Cyclic voltammetry and spectroelectrochemistry

**Fig. 4.**
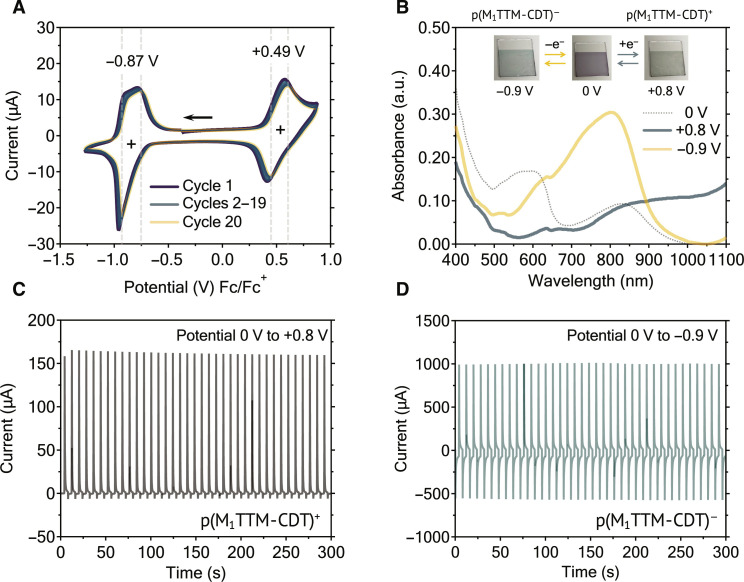
Electrochemical and electrochromic properties. (**A**) Cyclic voltammetry of p(M_1_TTM-CDT) measured in deoxygenated and anhydrous acetonitrile with 0.1 M TBAPF_6_ electrolyte against ferrocene, using glassy carbon and platinum as working and counter electrodes, respectively. (**B**) Film-state (spin-coated on ITO glass substrates) spectroelectrochemical spectra of p(M_1_TTM-CDT) at its neutral, oxidized, and reduced states. (**C** and **D**) Oxidation and reduction cycles of p(M_1_TTM-CDT) thin film.

We performed cyclic voltammetry to understand the electrochemical properties of these radicals. (M_2_TTM)_2_-CDT diradical shows stable and reversible redox processes, with a half-wave reduction potential at −1.21 V and a pair of sequential oxidation potentials at +0.17 and +0.67 V (fig. S14A), which correspond to the oxidation of radical sites and the CDT unit, respectively. p(M_1_TTM-CDT) exhibits a reduction potential at −0.87 V and an oxidation potential at +0.49 V (Fig. 4A), both of which are shifted relative to the redox potentials of the diradical, possibly due to the increased π-conjugation along the backbone. Note that (M_2_TTM)_2_-CDT exhibits two reversible shoulder-like reduction features at −1.17 and −1.23 V, and p(M_1_TTM-CDT) shows a single reversible shoulder peak at −0.92 V, which indicate non-negligible interactions between the unpaired electrons (*51*). In addition to the aforementioned photostability, both radical species demonstrate outstanding reversible redox processes, where no degradations of the peak currents are observed over 20 full redox cycles. These results indicate excellent photo- and electrochemical stabilities of the open-shell radical systems, making them promising candidates for a range of energy storage applications.

In light of the reversible redox processes, we carried out film-state spectroelectrochemical measurements for the radical materials. (M_2_TTM)_2_-CDT and p(M_1_TTM-CDT) exhibit distinct absorption features among the neutral, oxidized, and reduced states, with p(M_1_TTM-CDT) showing a noticeable three-color switching from neutral (purple) to oxidized (semitransparent green) and reduced (semitransparent blue) states (Fig. 4B). In both cases, λ_max_ of reduced states show a blue shift relative to that of the neutral state. While the oxidized diradical (M_2_TTM)_2_-CDT exhibits a λ_max_ of 960 nm (fig. S15A), with a localized polaronic feature, the polyradical p(M_1_TTM-CDT) demonstrates a broad absorption band feature in its oxidized state in the NIR region, suggesting that the charge carrier is quite delocalized within the system (Fig. 4B) (*52*). The film-state electrochromic behavior of p(M_1_TTM-CDT) is reversible on the indium tin oxide (ITO) glass substrate. We monitored the transmittance at 1100 nm for the oxidation and 800 nm for the reduction (fig. S15, B and C), while cycling from 0 to +0.8 V and 0 to −0.9 V, respectively, and the polyradical thin film can be effectively switched between the two states up to 500 and 60 cycles, respectively (Fig. 4, C and D, and fig. S15, D and E). The high stability and redox behaviors clearly demonstrate excellent potential of these materials for NIR electrochromic or energy storage applications (*53*, *54*).

## DISCUSSION

We have presented two open-shell TTM-based radical systems with a planar conjugated π-linker showing outstanding chemical, thermal, and electrochemical stabilities. These materials have strong panchromatic absorption across the entire visible spectrum and emission in the NIR region (~1000 nm) in both solution and film states. The NIR optical properties are a result of a zwitterionic excited state as the lowest-energy electronic transition. From EPR measurements, we demonstrate that the CDT linker allows for spin-spin couplings in the (M_2_TTM)_2_-CDT diradical, which are not observed in the polyradical p(M_1_TTM-CDT) due to more pronounced disordering and spin dynamics. In addition, SQUID studies suggest that considerable antiferromagnetic exchange is present in both derivatives, indicating their open-shell singlet ground state characters, which is also supported by computation. (M_2_TTM)_2_-CDT and p(M_1_TTM-CDT) are redox active, and the polyradical p(M_1_TTM-CDT) thin film demonstrates reversible electrochromic processes up to hundreds of cycles under ambient conditions. Our results suggest that conjugated polyradicals are promising functional materials for future photovoltaic, OLEDs, spintronic, and energy storage applications where their unique combination of NIR optical, unpaired spin correlations, and electrochromic properties could potentially surpass those of conventional closed-shell materials.

## MATERIALS AND METHODS

### Materials and general characterizations

1,3,5-Trichlorobenzene, anhydrous chloroform, dimethyl sulfoxide, tetrahydrofuran, and 1,4-dioxane were purchased from Acros Organics. 4*H*-cyclopenta[2,1-*b*:3,4-*b′*]dithiophene was obtained from Ossila, and all other reagents used in the synthesis were purchased from Sigma-Aldrich and used as received. Flash chromatography was carried out using Biotage Isolera Four System and Biotage Sfär Silica flash cartridges. ^1^H and ^13^C NMR spectra were recorded on 400-MHz Avance III HD and 500-MHz Avance III Spectrometers. Chemical shifts were reported in parts per million (ppm, d scale) from residual protons in the deuterated solvent for ^1^H [7.26 ppm for chloroform-*d* (CDCl_3_) and 5.32 ppm for dichloromethane-*d*_2_ (CD_2_Cl_2_)] and ^13^C NMR [77.16 ppm for chloroform-*d* (CDCl_3_) and 54.00 ppm for dichloromethane-*d*_2_ (CD_2_Cl_2_)]. The data were presented in the following format: chemical shift, multiplicity (s = singlet, d = doublet, t = triplet, quint = quintet, m = multiplet, br = broad, brm = broad multiplet), coupling constant in hertz (Hz), signal area integration in natural numbers. Mass spectra were obtained using a Waters Xevo G2-S benchtop QTOF mass spectrometer (equipped with an atmospheric solid analysis probe) in Yusuf Hamied Department of Chemistry, University of Cambridge. C, H, and N combustion elemental analyses were obtained on an Exeter Analytical Inc. CE-440 elemental analyzer, and the results are reported as an average of two samples. Gel permeation chromatography (GPC) was carried out using an Agilent 1200 Series GPC-SEC System equipped with two sequential Phenogel 10 μm Linear (2) 300 mm by 7.8 mm Liquid Chromatography (LC) columns. The eluent was chlorobenzene, and the operating temperature was 80°C. The number-average (*M*_n_) and weight-average (*M*_w_) molecular weights were determined against polystyrene standards.

### UV-vis and photoluminescence spectroscopy

Solution- and film-state UV-vis spectra were measured with a Shimadzu UV-1800 spectrophotometer. Photoluminescence was measured in a home-built setup by providing a continuous wave excitation at 532 nm using a diode laser. Photoluminescence is collected in a reflection mode setup after passing photons through a 550-long-pass filter (Thorlabs). The transmitted photons then are collected in a collimating two-lens apparatus and directed into an optical fiber which supplies the photons into a calibrated grating spectrometer (Andor SR-303i) and lastly into a Si camera where it is recorded. Output spectra are corrected taking into account the filter transmission and camera sensitivity.

### Photoluminescence quantum efficiency

Steady-state PLQE measurements were performed using an integrating sphere. A continuous-wave 532-nm excitation is provided by a 532-nm diode laser with excitation powers of 10 to 300 mW cm^−2^. A focused beam of diameter 700 μm was used to excite the samples. The emission was directed using an optical fiber in a calibrated grating spectrometer (Andor SR-303i) onto a Si camera.

### Transient absorption spectroscopy

Transient absorption experiments were conducted on a setup pumped by a regenerative Ti:sapphire amplifier (Solstice Ace, Spectra-Physics) emitting 100-fs pulses centered at 800 nm at a rate of 1 kHz and a total output of 7 W. Depending on the probed spectral range and timescales, different combinations of optical systems were used.

### Cyclic voltammetry

Cyclic voltammetry was carried out on a PalmSens EmStat4S potentiostat in a three-electrode setup using a glassy carbon electrode (3.0-mm diameter) as the working electrode, platinum wire as the counter electrode, and freshly activated silver wire as the Ag/Ag^+^ reference electrode. The silver wire was activated by immersing in concentrated HCl solution to remove any silver oxides or other impurities and then rinsed with water and acetone and dried before each measurement. The reference electrode was calibrated against ferrocene/ferrocenium (Fc/Fc^+^) redox couple at the end of each measurement (the Fc/Fc^+^ half-wave potential, *E*_1/2_, was determined at 0.20 V versus Ag/Ag^+^). The supporting electrolyte was 0.1 M solution of Bu_4_NPF_6_ in anhydrous tetrahydrofuran or acetonitrile, and the scan rate was 0.1 V s^−1^.

### Spectroelectrochemistry

Electrochromic properties were simultaneously measured with a Shimadzu UV-1800 and a PalmSens EmStat4S potentiostat. A solution of p(M_1_TTM-CDT) (5.0 mg/ml in toluene) was spin-coated (2000 rpm for 45 s) on a commercially available ITO-coated glass slide (surface resistivity, 8 to 12 ohm/square) from Sigma-Aldrich. The p(M_1_TTM-CDT)–coated ITO substrate was used as the working electrode, platinum wire was the counter electrode, and freshly activated silver wire was the Ag/Ag^+^ reference electrode. The supporting electrolyte was 0.1 M solution of Bu_4_NPF_6_ in acetonitrile, and the scan rate was 0.1 V s^−1^.

### Computational details

Computations of the excited states were performed at the MS-CASPT2/ANO-S-VDZP level of theory (*34*, *55*) in OpenMolcas (*56*) using an ionization potential/electron affinity (IPEA) shift of 0.25. The computations were performed in C_s_ symmetry with three a′ and three a″ orbitals in the active space. State averaging was performed over three states each of ^1^A′, ^1^A″, ^3^A′, and ^3^A″ symmetries, and the same states were used in the multistate CASPT2 procedure. The geometries were optimized in their triplet states at the r2-SCAN-3c level as implemented in Orca 5.0.1 (*57*).

### EPR spectroscopy

All EPR measurements were conducted using a Bruker E500 spectrometer equipped with an X-band microwave source and Bruker’s ER 4122SHQE cavity. Various temperature (290 to 5 K) measurements were carried out with an Instrument ESR900 helium cryostat, controlled with a MercuryiTC temperature controller. For sample preparation, all samples were dissolved in toluene at a concentration of 50 μM and then transferred into glass capillaries. All spectra showed in this work were measured with a microwave power of 50 mW. EasySpin software (*58*) was used to simulate the EPR spectra.

### Superconducting quantum interference device

Magnetization measurements were obtained in a Quantum Design Magnetic Properties Measurement System (MPMS 3) using a SQUID magnetometer. Measurements were performed down to 1.8 K and up to 350 K, in various applied magnetic field strengths up to 7 T. Each magnetization-temperature curve was measured starting at 1.8 K and increasing the temperature in a constant probing field of 50 mT. Before beginning any new magnetic field or temperature scan, the system was taken to 300 K, and the magnet was reset to remove any stray flux from the SQUID. Samples were secured on an MPMS quartz sample holder using GE varnish, and care was taken to ensure that they were not touched by any magnetic material throughout the mounting and loading procedure. At each measurement, several dc magnetization measurements were averaged, providing a reliable measure of the bulk magnetization of the samples. For both samples, 8 mg was added into a plastic capsule. The dc moment was averaged over multiple scans, and between each experimental run, the MPMS was brought to 300 K, and the magnet was reset to remove any remaining flux in the SQUID.

## Supplementary Materials

### This PDF file includes:

Figs. S1 to S15 Table S1 to S6 References
