# Supplementary material for: Near-infrared luminescent open-shell π-conjugated systems with a bright lowest-energy zwitterionic singlet excited state
Source: Sci Adv. 2024 Jul 24;10(30):eado3476. doi: 10.1126/sciadv.ado3476 (PMC11268402; doi:10.1126/sciadv.ado3476)
Supplement: Supplementary file 1 — Figs. S1 to S15 Table S1 to S6 References [file sciadv.ado3476_sm.pdf]

Science Advances
AAAS

Supplementary Materials for

Near-infrared luminescent open-shell π-conjugated systems with a bright lowest-energy zwitterionic singlet excited state

Craig P. Yu *et al.*

Corresponding author: Hugo Bronstein, hab60@cam.ac.uk; Richard H. Friend, rhf10@cam.ac.uk; Petri Murto, pm707@cam.ac.uk

Sci. Adv. **10**, eado3476 (2024)
DOI: 10.1126/sciadv.ado3476

This PDF file includes:

Supplementary Text
Figs. S1 to S15
Tables S1 to S6
References

Materials and General Characterization Methods

^1H and ^{13}C NMR spectra were recorded on 400 MHz Avance III HD and 500 MHz Avance III Spectrometers. Chemical shifts were reported in parts per million (ppm, d scale) from residual protons in the deuterated solvent for ^1H (7.26 ppm for chloroform-*d* ($CDCl_3$) and 5.32 ppm for dichloromethane-d_2 (CD_2Cl_2)) and ^{13}C NMR (77.16 ppm for chloroform-*d* ($CDCl_3$) and 54.00 ppm for dichloromethane-d_2 (CD_2Cl_2)). The data were presented in the following format: chemical shift, multiplicity (s = singlet, d = doublet, t = triplet, quint = quintet, m = multiplet, br = broad, brm = broad multiplet), coupling constant in Hertz (Hz), signal area integration in natural numbers. Mass spectra were obtained using a Waters Xevo G2-S benchtop QTOF mass spectrometer (equipped with an atmospheric solids analysis probe, ASAP) in Yusuf Hamied Department of Chemistry, University of Cambridge. C, H, N combustion elemental analyses (EA) were obtained on an Exeter Analytical Inc. CE-440 elemental analyser and the results are reported as an average of two samples. Gel permeation chromatography (GPC) was carried out using an Agilent 1200 Series GPC-SEC System equipped with two sequential Phenogel™ 10 µm Linear(2) 300 × 7.8 mm LC columns. The eluent was chlorobenzene and the operating temperature was 80 °C. The number-average (M_n) and weight-average (M_w) molecular weights were determined against polystyrene standards. Thermal gravimetric analysis (TGA) was run under N_2 atmosphere at a heating rate of 10 °C/min using a Mettler Toledo TGA/DSC instrument at a gas flow of 100 cm^3/min. Density functional theory calculations were performed using the Gaussian 16 program (*59*) and the initial geometries were constructed in GaussView. Ground state geometries were optimized at the UB3LYP/def2-SVP level.

Design and Synthesis

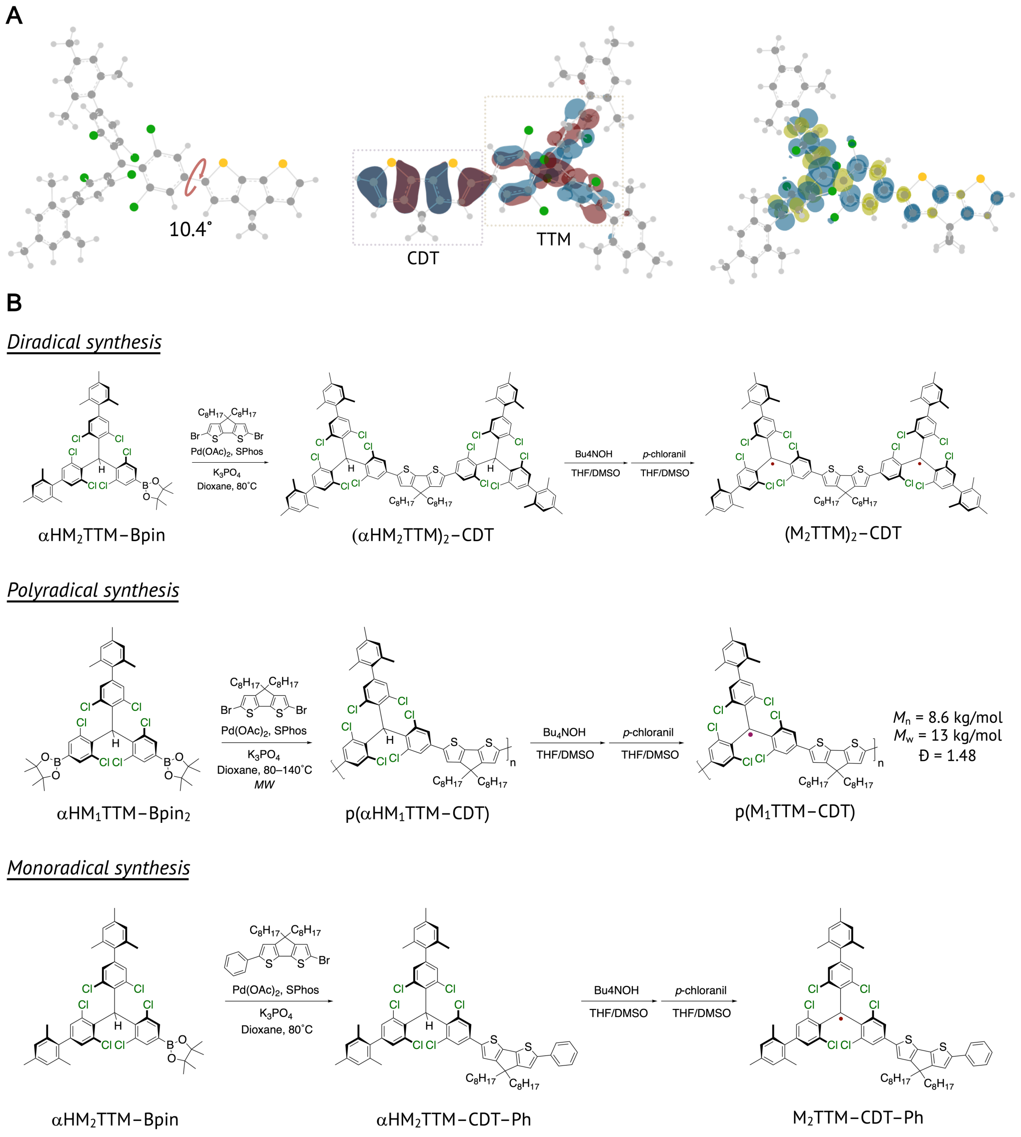

Fig. S1.

Design and synthesis of $(M_2TTM)_2$–CDT and p(M_1TTM–CDT). (**A**) Optimized structure, HOMO distribution, and spin density (blue: spin up, yellow: spin down) of one repeat unit of p(M_1TTM–CDT) (with two mesityl groups) calculated at the UB3LYP/def2-SVP level of theory. (**B**) Synthetic schemes of $(M_2TTM)_2$–CDT, p(M_1TTM–CDT), and M_2TTM–CDT–Ph.

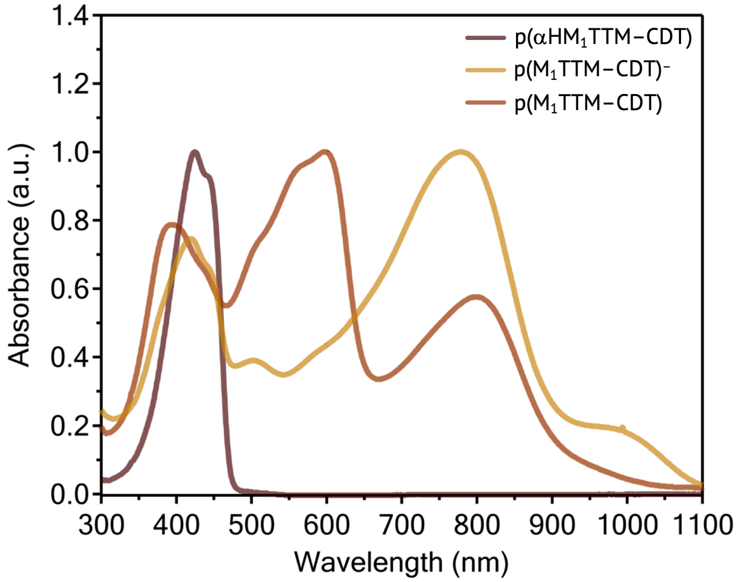

Fig. S2.

Polyradical conversion monitoring. UV-vis absorption spectra of p(αHM$_1$TTM–CDT), anionic p(M$_1$TTM–CDT)$^-$, and the target p(M$_1$TTM–CDT).

Synthetic Procedures

Synthesis of tris(2,4,6-trichlorophenyl)methane (αHTTM)

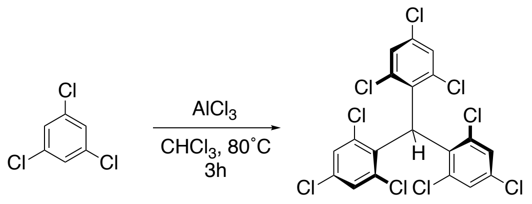

1,3,5-trichlorobenzene (82.1 g, 453 mmol, 9.00 equiv.) was added into a 500 mL pressure flask, which was purged with Ar gas. Anhydrous $AlCl_3$ (7.32 g, 55.3 mmol, 1.10 equiv.) was added, followed by anhydrous $CHCl_3$ (4.03 mL, 50.2 mmol, 1.00 equiv.). The flask was sealed and heated at 80˚C for 3 h. After cooling to room temperature, the flask was carefully opened to release the generated HCl gas. The solidified reaction mixture was quenched by H_2O and extracted with $CHCl_3$, and the organic layer was dried over Na_2SO_4. Solvent was removed *in vacuo* and the crude product was recrystallised from a mixture of hexane/DCM to give the target compound as a white solid (21.6 g, 70% yield). ^1H NMR (400 MHz, $CDCl_3$) δ 7.36 (d, $J = 2.2$ Hz, 3H), 7.23 (d, $J = 2.2$ Hz, 3H), 6.68 (s, 1H). The spectrum is in good agreement with the reported data (*14*).

Synthesis of αHM₁TTM and αHM₂TTM

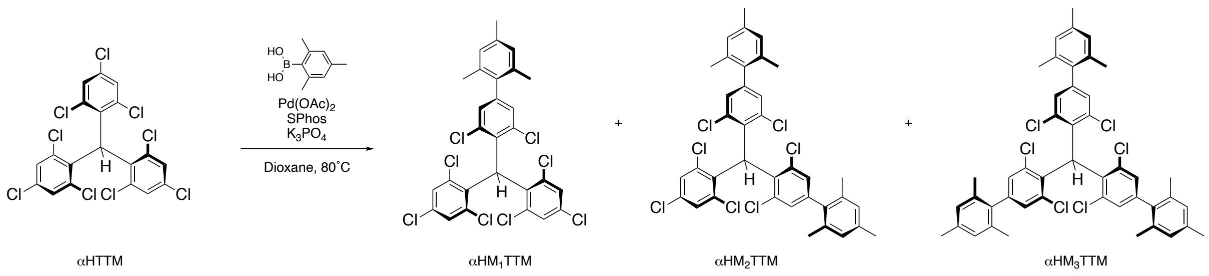

αHTTM (5.00 g, 9.02 mmol, 1.00 equiv.), mesitylboronic acid (2.22 g, 13.5 mmol, 1.50 equiv.), $Pd(OAc)_2$ (0.0405 g, 0.180 mmol, 2 mol%.), SPhos (0.148 g, 0.361 mmol, 4 mol%) and K_3PO_4 (9.19 g, 43.3 mmol, 4.80 equiv.) were charged into a three-neck round bottom flask equipped with a condenser and the reaction vessel was subjected to three vacuum/Ar gas refill cycles. Anhydrous 1,4-dioxane (56.4 mL, 0.160 M) was added and the mixture was heated at 80˚C for 24 h. After cooling to temperature, the mixture was quenched with water and extracted with dichloromethane, and the organic phase was dried over Na_2SO_4. Solvent was removed *in vacuo* and the crude product was purified with column chromatography, by gradually increasing the eluent polarity from hexane (fractions 1 and 2) to 2% (v/v) dichloromethane/hexane (fraction 3), and finally to 5% (v/v) dichloromethane/hexane (fraction 4). Fraction 1 was the unreacted starting material αHTTM (0.705 g, 14% yield). The target compounds αHM₁TTM and αHM₂TTM were obtained as fractions 2 and 3, respectively.

4'-(Bis(2,4,6-trichlorophenyl)methyl)-3',5'-dichloro-2,4,6-trimethyl-1,1'-biphenyl (αHM$_1$TTM)

Target compound was collected as fraction 2 as a crystalline white solid (2.73 g, 47% yield). ^{1}H NMR (400 MHz, $CDCl_3$) δ 7.38 (dd, J = 3.8, 2.1 Hz, 2H), 7.25 (t, 2H), 7.14 (d, J = 1.7 Hz, 1H), 7.01 (d, J = 1.8 Hz, 1H), 6.93 (s, 2H), 6.80 (s, 1H), 2.32 (s, 3H), 2.02 (d, J = 4.3 Hz, 6H). The spectrum is in good agreement with the reported data (*14*).

4',4'''-((2,4,6-Trichlorophenyl)methylene)bis(3',5'-dichloro-2,4,6-trimethyl-1,1'-biphenyl) (αHM$_2$TTM)

Target compound was collected as fraction 3 as white solid (1.27 g, 20% yield). ^{1}H NMR (400 MHz, $CDCl_3$) δ 7.40 (d, J = 2.3 Hz, 1H), 7.27 (d, J = 2.3 Hz, 1H), 7.16 (dd, J = 6.2, 1.8 Hz, 2H), 7.03 (t, J = 2.1 Hz, 2H), 6.94 (s, 4H), 6.91 (s, 1H), 2.33 (s, 6H), 2.05 (dd, J = 4.7, 3.2 Hz, 12H). The spectrum is in good agreement with the reported data (*14*).

Synthesis of 2-(4-(Bis(3,5-dichloro-2',4',6'-trimethyl-[1,1'-biphenyl]-4-yl)methyl)-3,5-dichlorophenyl)-4,4,5,5-tetramethyl-1,3,2-dioxaborolane (M_2TTM-Bpin)

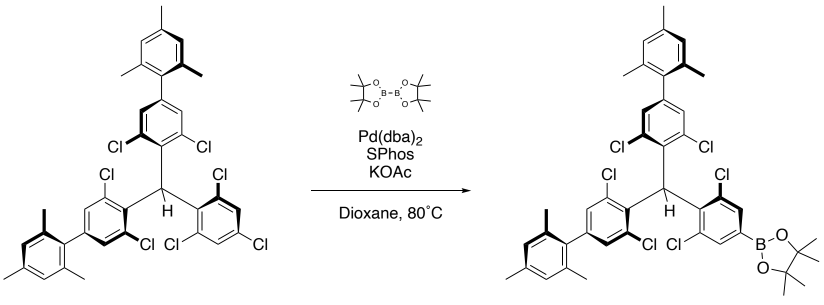

αHM_2TTM (808 mg, 1.12 mmol, 1.00 equiv.), bis(pinacolato)diboron (427 mg, 1.68 mmol, 1.50 equiv.), Pd(dba)$_2$ (5.03 mg, 0.0224 mmol, 2 mol%), SPhos (23.0 mg, 0.0560 mmol, 5 mol%) and KOAc (0.245 g, 2.02 mmol, 1.80 equiv.) were added into a flame-dried Schlenk tube under Ar. Anhydrous 1,4-dioxane (11.2 mL, 0.100 M) was added and the mixture was heated at 80˚C for 24 h. After cooling to room temperature, the mixture was quenched with H_2O and extracted with dichloromethane, and the organic phase was dried over Na_2SO_4. Solvent was removed *in vacuo* and the crude product was purified with column chromatography, by gradually increasing the eluent polarity from hexane to 5% (v/v) DCM in hexane (before loading the crude product, the column was pre-treated by passing through a mixture of 3% (v/v) acetic acid in hexane until the silica was saturated with acetic acid, then excess acetic acid was washed out by flushing hexane through the column). The target compound was collected as a white solid (670 mg, 74% yield). ^{1}H NMR (400 MHz, $CDCl_3$) δ 7.77 (d, J = 1.2 Hz, 1H), 7.64 (d, J = 1.3 Hz, 1H), 7.14 (dd, J = 11.6, 1.8 Hz, 2H), 7.01 (dd, J = 7.9, 1.8 Hz, 2H), 6.96 (s, 1H), 6.93 (s, 4H), 2.32 (s, 6H), 2.04 (dd, J = 6.9, 3.0 Hz, 12H), 1.34 (d, J = 5.2 Hz, 12H). The spectrum is in good agreement with the reported data (*14*).

Synthesis of 2,2'-(((3,5-Dichloro-2',4',6'-trimethyl-[1,1'-biphenyl]-4-yl)methylene)bis(3,5-dichloro-4,1-phenylene))bis(4,4,5,5-tetramethyl-1,3,2-dioxaborolane) (M_1TTM-Bpin$_2$)

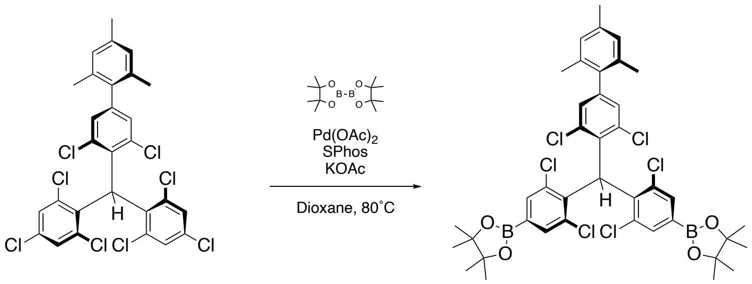

αHM$_1$TTM (2.38 g, 3.733 mmol, 1.00 equiv.), bis(pinacolato)diboron (2.84 mg, 11.2 mmol, 3.00 equiv.), Pd(dba)$_2$ (0.0168 g, 0.0747 mmol, 2 mol%), SPhos (0.0766 mg, 0.187 mmol, 5 mol%) and KOAc (1.32 g, 13.4 mmol, 3.60 equiv.) were added into a flame-dried Schlenk tube under Ar. Anhydrous 1,4-dioxane (37.3 mL, 0.100 M) was added and the mixture was heated at 80˚C for 24 h. After cooling to room temperature, the mixture was quenched with H_2O and extracted with dichloromethane, and the organic phase was dried over Na_2SO_4. Solvent was removed *in vacuo* and the crude product was dissolved in a mixture of dichloromethane/hexane and filtered through a short pad of celite. The target compound was collected as a white solid (2.63 g, 86% yield). ^{1}H NMR (400 MHz, $CDCl_3$) δ 7.74 (dd, J = 10.7, 1.3 Hz, 2H), 7.61 (dd, J = 9.7, 1.3 Hz, 2H), 7.10 (d, J = 1.8 Hz, 1H), 6.98 (d, J = 1.7 Hz, 1H), 6.92 (d, J = 2.1 Hz, 3H), 2.31 (s, 3H), 2.02 (d, J = 3.4 Hz, 6H), 1.34 (d, J = 3.4 Hz, 24H). The spectrum is in good agreement with the reported data (*14*).

Synthesis of 4,4-dioctyl-4H-cyclopenta[2,1-b:3,4-b']dithiophene

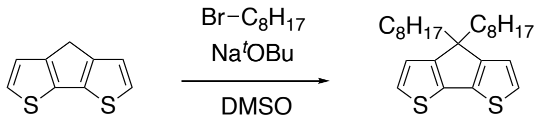

To a 100 mL round-bottom flask that is flushed with Ar was charged 4*H*-cyclopenta[2,1-*b*:3,4-*b'*]dithiophene (1.50 g, 8.41 mmol, 1.00 equiv.), Na^{t}OBu (3.23 g, 33.6 mmol, 4.00 equiv.), and anhydrous dimethyl sulfoxide (42.1 mL, 0.20 M), and the mixture was heated at 50˚C under Ar. Then, 1-bromooctane (6.43 mL, 21.0 mmol, 2.49 equiv.) was added dropwise via a syringe and the mixture was stirred at 50˚C overnight. The reaction mixture was quenched with H_2O and extracted with Et_2O, and the organic layer was dried over Na_2SO_4. The solvent was removed *in vacuo*, and the crude was purified by column chromatography using hexane as the eluent to give the target compound as a yellow oil (3.11 g, 91% yield). ^{1}H NMR (400 MHz, $CDCl_3$) δ 7.14 (d, 2H), 6.93 (d, 2H), 1.81 (t, 4H), 1.35–1.00 (m, 24H), 1.00–0.89 (m, 4H), 0.84 (t, 6H). The spectrum is in good agreement with the reported data (*60*).

Synthesis of 2,6-dibromo-4,4-dioctyl-4H-cyclopenta[2,1-b:3,4-b']dithiophene (C_8–CDT–Br_2)

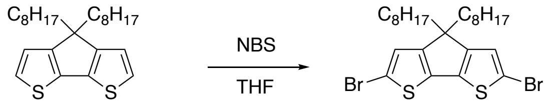

To a 500 mL three-neck round bottom flask was charged 4,4-dioctyl-4*H*-cyclopenta[2,1-*b*:3,4-*b'*]dithiophene (4.50 g, 11.2 mmol, 1.00 equiv.) and anhydrous tetrahydrofuran (112 mL, 0.100 M) under an atmosphere of Ar. Then, *N*-bromosuccinimide (4.18 g, 23.5 mmol, 2.10 equiv.) was added into the solution portion-wise at 0˚C, and the mixture was slowly warmed to room temperature and stirred in the dark for 12 h. The reaction mixture was quenched with H_2O and extracted with Et_2O, and the organic layer was dried over Na_2SO_4. The solvent was removed *in vacuo*, and the crude was purified by column chromatography using hexane as the eluent to give the target compound as a yellow oil (5.89 g, 94% yield). ^{1}H NMR (400 MHz, $CDCl_3$) δ 6.92 (s, 2H), 1.75 (quint, J = 4.0, 4.0, 4.0, 4.0 Hz, 4H), 1.28-1.13 (m, 18H), 0.94–0.84 (m, 12H). The spectrum is in good agreement with the reported data (*61*).

Synthesis of 2,6-bis(4-(bis(3,5-dichloro-2',4',6'-trimethyl-[1,1'-biphenyl]-4-yl)methyl)-3,5-dichlorophenyl)-4,4-dioctyl-4H-cyclopenta[2,1-b:3,4-b']dithiophene ((αHM$_2$TTM)$_2$–CDT)

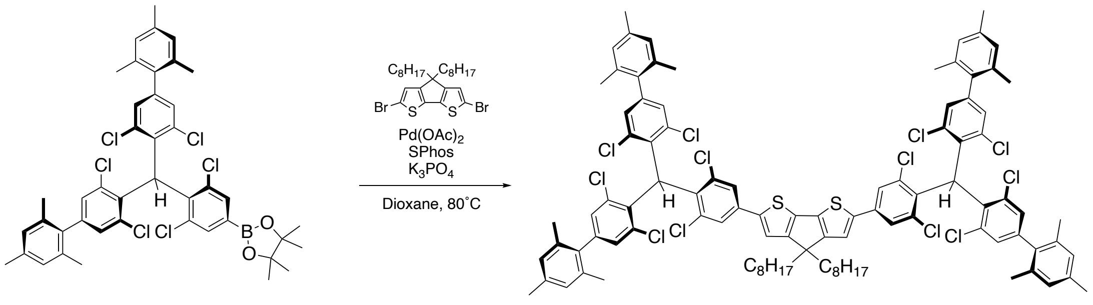

αHM$_2$TTM–Bpin$_2$ (565.92 mg, 0.696 mmol, 2.00 equiv.), C_8–CDT–Br_2 (195 mg, 0.348 mmol, 1.00 equiv.), $Pd(OAc)_2$ (2.34 mg, 0.0104 mmol, 3 mol%), SPhos (10.7 mg, 0.0261 mmol, 7.5 mol%) and K_3PO_4 (295 mg, 1.39 mmol, 4.00 equiv.) were added into a flame-dried Schlenk tube under Ar. Anhydrous 1,4-dioxane (3.50 mL, 0.100 M) was added and the mixture was heated at 80˚C for 24 h. After cooling to room temperature, the mixture was quenched with H_2O and extracted with dichloromethane, and the organic phase was dried over Na_2SO_4. Solvent was removed *in vacuo* and the crude product was purified with column chromatography, by gradually increasing the eluent polarity from hexane to 3% (v/v) EtOAc in hexane. The target compound was collected as a yellow solid (839.1 mg, 68% yield). ^{1}H NMR (400 MHz, $CDCl_3$) δ 7.66 (d, J = 1.8 Hz, 2H), 7.52 (d, J = 2.1 Hz, 2H), 7.31 (s, 2H), 7.19 (dd, J = 8.0, 4.0 Hz, 4H), 7.06 (t, J = 2.1, 2.4, 4H), 6.97 (s, 2H), 6.94 (s, 8H), 2.31 (s, 12H), 2.05 (s, 24H), 1.94-1.89 (m, 4H), 1.28 – 1.13 (m, 20H), 0.98 (s, 4H), 0.83 (t, J = 8.0, 8.0 Hz, 6H). ^{13}C NMR (101 MHz, CD_2Cl_2) δ 159.93, 142.70, 141.74, 138.36, 137.83, 137.74, 137.14, 137.11, 136.37, 136.18, 135.99, 135.90, 134.70, 134.41, 134.31, 131.65, 131.55, 129.87, 128.53, 126.62, 124.90, 119.63, 54.84, 51.00, 38.25, 32.22, 30.37, 29.66, 24.91, 23.06, 21.18, 20.72, 14.27. TOF-MS-ES$^+$ Calcd. for $[C_{99}H_{94}Cl_{12}S_2]^+$: 1767.3221. Found: m/z = 1767.3138. EA Calcd. for $C_{99}H_{94}Cl_{12}S_2$: C, 67.05; H, 5.34. Found: C, 66.90; H, 5.31.

Synthesis of poly[4,4-dioctyl-4H-cyclopenta[2,1-b:3,4-b']dithiophene -2,6-diyl-alt-4'-(bis(2,6-dichlorophenyl)methyl)-3',5'-dichloro-2,4,6-trimethyl-1,1'-biphenyl-4,4'-diyl] (p(αHM$_1$TTM–CDT))

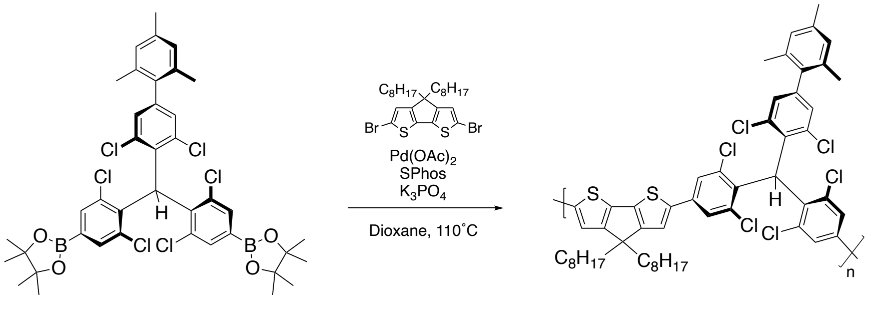

αHM$_1$TTM–Bpin$_2$ (303 mg, 0.370 mmol, 1.00 equiv.), C_8–CDT–Br_2 (207 mg, 0.370 mmol, 1.00 equiv.), Pd(OAc)$_2$ (0.830 mg, 0.00370 mmol, 1 mol%), SPhos (3.04 mg, 0.00740 mmol, 2 mol%) and K_3PO_4 (314 mg, 1.48 mmol, 4.00 equiv.) were added into a flame-dried Schlenk tube under Ar. Anhydrous 1,4-dioxane (3.70 mL, 0.100 M) was added and the mixture was heated at 110˚C for 72 h. After cooling to room temperature, the mixture was added to methanol dropwise and the precipitates were collected by vacuum filtration. The brown solids were subjected to three cycles of Soxhlet extraction using methanol, hexane, and acetone as solvents, and the polymer was eventually collected from the chloroform fraction. The chloroform fraction was concentrated *in vacuo* and slowly added to methanol, the precipitates were collected via vacuum filtration to afford the target polymer as a deep yellow solid (277 mg, 75% yield). GPC: M_n = 8.6 kg/mol, M_w = 13 kg/mol, $Đ$ = 1.48. ^{1}H NMR (400 MHz, $CDCl_3$) δ 7.61 (s, 2H), 7.48 (s, 2H), 7.23 (s, 2H), 7.15 (s, 1H), 7.03 (s, 1H), 6.93 (s, 3H), 6.90 (s, 1H), 6.09 (s, 4H), 2.32 (s, 3H), 2.05 (s, 6H), 1.87 (s, 3H), 1.23 (s, 4H), 1.16 (s, 14H), 0.96 (s, 3H), 0.83 (s, 6H). EA Calcd. for $C_{55}H_{60}Cl_6S_2$: C, 66.20; H, 6.06. Found: C, 66.23; H, 6.07.

Synthesis of 2-bromo-4,4-dioctyl-4H-cyclopenta[2,1-b:3,4-b']dithiophene

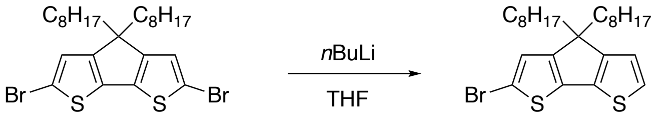

To a flame-dried Schlenk tube was added 2,6-dibromo-4,4-dioctyl-4*H*-cyclopenta[2,1-*b*:3,4-*b'*]dithiophene (540 mg, 0.964 mmol, 1.00 equiv.) and anhydrous tetrahydrofuran (12.0 mL, 0.08 M). After cooling the solution to -78˚C, *n*-butyllithium (1.60 M in hexane, 0.964 mmol, 1.00 equiv.) was added dropwise under Ar. The reaction mixture was stirred for 0.5 h at the same temperature and quenched with H_2O. The crude was extracted with hexane and the organic layer was dried over Na_2SO_4 and concentrated *in vacuo*. The deep-yellow oil was purified with column chromatography using hexane as the eluent to give the target compound as a light-yellow oil (447 mg, 97% yield). ^{1}H NMR (400 MHz, $CDCl_3$) δ 7.16 (s, 1H), 6.94 (s, 1H), 6.91 (d, J = 4.8 Hz, 1H), 1.78 (t, J = 7.6, 7.6 Hz, 4H), 1.27-1.10 (m, 20H), 0.95-0.82 (m, 10H). ^{13}C NMR (126 MHz, CDCl3) δ 157.47, 156.86, 137.03, 136.18, 125.13, 124.85, 121.69, 110.56, 54.30, 37.77, 31.95, 30.12, 29.47, 29.39, 24.61, 22.77, 14.24. TOF-MS-ASAP$^+$ Calcd. for $[C_{25}H_{37}BrS_2]^+$: 481.1598. Found: *m/z* = 481.1578.

Synthesis of 4,4-dioctyl-2-phenyl-4H-cyclopenta[2,1-b:3,4-b']dithiophene

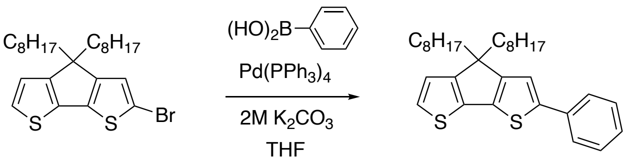

To a flame-dried Schlenk tube was charged 2-bromo-4,4-dioctyl-4H-cyclopenta[2,1-b:3,4-b']dithiophene (360 mg, 0.748 mmol, 1.00 equiv.), phenylboronic acid (95.7 mg, 0.785 mmol, 1.05 equiv.), tetrakis(triphenylphosphine)palladium(0) (17.3 mg, 0.0150 mmol, 2.00 mol%) under Ar. To this reaction vessel was then added a mixture of degassed aqueous 2 M K_2CO_3 (0.560 mL) and tetrahydrofuran (2.50 mL, 0.300 M), and the reaction mixture was heated at 80˚C for 10 h. After cooling to room temperature, diethyl ether and H_2O were added, and the organic layer was dried over Na_2SO_4 and concentrated *in vacuo*. The crude was purified with column chromatography using hexane as the eluent to give the target compound as a light-yellow oil (320 mg, 89% yield). ^1H NMR (400 MHz, CD_2Cl_2) δ 7.62 (d, J = 7.2 Hz, 2H), 7.38 (t, J = 7.7, 8.0 Hz, 2H), 7.29 – 7.18 (m, 3H), 6.98 (d, J = 4.8 Hz, 1H), 1.87 (t, J = 9.0, 7.6 Hz, 4H), 1.28–1.16 (m, 20H), 1.01-0.95 (m, 4H), 0.84 (t, J = 6.8, 7.2 Hz, 6H). ^{13}C NMR (101 MHz, CD_2Cl_2) δ 159.07, 158.06, 143.87, 136.45, 135.92, 135.22, 128.90, 126.98, 125.01, 124.92, 121.71, 117.94, 53.77, 37.75, 31.81, 30.01, 29.35, 29.24, 24.54, 22.62, 13.83. TOF-MS-ASAP$^+$ Calcd. for $[C_{31}H_{42}S_2]^+$: 479.2806. Found: m/z = 479.2807.

Synthesis of 2-bromo-4,4-dioctyl-6-phenyl-4H-cyclopenta[2,1-b:3,4-b']dithiophene

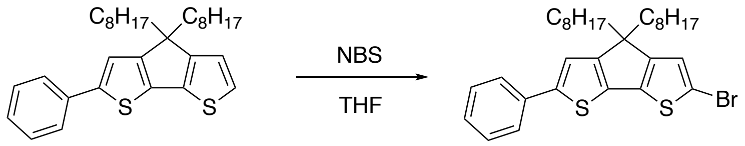

To a flame-dried Schlenk tube was charged 4,4-dioctyl-2-phenyl-4H-cyclopenta[2,1-b:3,4-b']dithiophene (177 mg, 0.369 mmol, 1.00 equiv.) and anhydrous tetrahydrofuran (7.50 mL, 0.05 M) under an atmosphere of Ar. Then, *N*-bromosuccinimide (68.9 g, 0.387 mmol, 1.05 equiv.) was added into the solution portion-wise at 0˚C, and the mixture was slowly warmed to room temperature and stirred in the dark for 12 h. The reaction mixture was quenched with H_2O and extracted with Et_2O, and the organic layer was dried over Na_2SO_4. The solvent was removed *in vacuo*, and the crude was purified by column chromatography using hexane as the eluent to give the target compound as a yellow oil (195 mg, 95% yield). ^1H NMR (500 MHz, CD_2Cl_2) δ 7.61 (d, *J* = 8.4 Hz, 2H), 7.38 (t, *J* = 7.8 Hz, 2H), 7.29 – 7.21 (m, 2H), 7.00 (s, 1H), 1.85 (q, *J* = 5.0, 5.0, 5.0 Hz, 4H), 1.32 – 1.14 (m, 20H), 0.99–0.94 (m, 4H), 0.84 (t, *J* = 10, 5.0 Hz, 6H). ^{13}C NMR (101 MHz, CD_2Cl_2) δ 158.72, 157.02, 137.44, 135.76, 135.46, 129.37, 127.61, 125.51, 125.31, 118.19, 55.13, 38.10, 32.24, 32.03, 30.40, 29.76, 29.68, 24.91, 23.09, 23.06, 14.31, 14.27. TOF-MS-ASAP$^+$ Calcd. for $[C_{31}H_{41}BrS_2]^+$: 557.1911. Found: *m/z* = 557.1907.

Synthesis of 2-(4-(bis(3,5-dichloro-2',4',6'-trimethyl-[1,1'-biphenyl]-4-yl)methyl)-3,5-dichlorophenyl)-4,4-dioctyl-6-phenyl-4H-cyclopenta[2,1-b:3,4-b']dithiophene (αHM₂TTM–CDT–Ph)

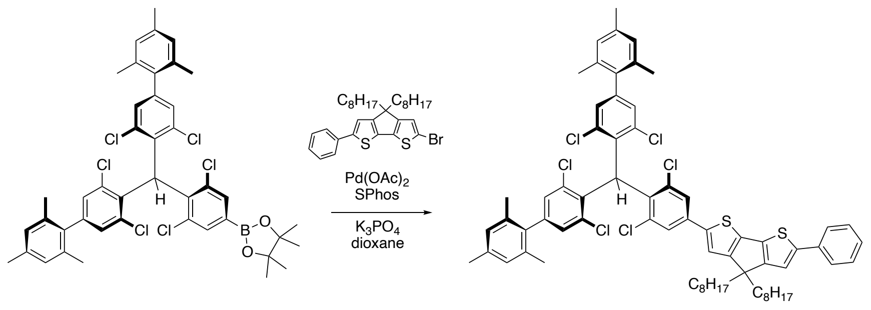

αHM_2TTM–$Bpin_2$ (135.00mg, 0.696 mmol, 2.00 equiv.), 2-bromo-4,4-dioctyl-6-phenyl-4H-cyclopenta[2,1-b:3,4-b']dithiophene (195 mg, 0.348 mmol, 1.00 equiv.), $Pd(OAc)_2$ (2.34 mg, 0.0104 mmol, 3 mol%), SPhos (10.7 mg, 0.0261 mmol, 7.5 mol%) and K_3PO_4 (295 mg, 1.39 mmol, 4.00 equiv.) were added into a flame-dried Schlenk tube under Ar. Anhydrous 1,4-dioxane (3.50 mL, 0.100 M) was added and the mixture was heated at 80˚C for 24 h. After cooling to room temperature, the mixture was quenched with H_2O and extracted with dichloromethane, and the organic phase was dried over Na_2SO_4. The crude product was purified with column chromatography, by gradually increasing the eluent polarity from hexane to 3% (v/v) EtOAc in hexane. The target compound was collected as a yellow solid (839.1 mg, 68% yield). ^1H NMR (500 MHz, CD_2Cl_2) δ 7.70 – 7.60 (m, 3H), 7.53 (d, J = 2.0 Hz, 1H), 7.40 (t, J = 7.8 Hz, 2H), 7.33 – 7.24 (m, 3H), 7.20 (dd, J = 5.3, 1.7 Hz, 2H), 7.09 – 7.04 (m, 2H), 6.98 (s, 1H), 6.95 (s, 4H), 2.32 (s, 6H), 2.06 (s, 12H), 1.92 (m, 4H), 1.26 – 1.14 (m, 20H), 1.02 (s, 4H), 0.84 (td, J = 7.0, 2.6 Hz, 6H). ^{13}C NMR (126 MHz, CD_2Cl_2) δ 160.04, 159.20, 145.79, 142.67, 140.77, 138.33, 138.24, 137.81, 137.71, 137.14, 136.38, 135.99, 135.92, 135.44, 134.45, 134.35, 131.64, 131.55, 129.87, 129.39, 128.52, 127.68, 126.50, 125.54, 124.78, 119.68, 118.25, 54.75, 51.00, 38.25, 32.24, 30.43, 29.69, 24.96, 23.06, 21.20, 20.68, 14.28. TOF-MS-ASAP⁻ Calcd. for $[C_{68}H_{70}Cl_6S_2]^-$: 1159.2972. Found: *m/z* = 1159.2966. EA Calcd. for $C_{68}H_{70}Cl_6S_2$: C, 70.16; H, 6.06. Found: C, 69.80; H, 5.75.

Synthesis of $(M_2TTM)_2$–CDT diradical via radical conversion

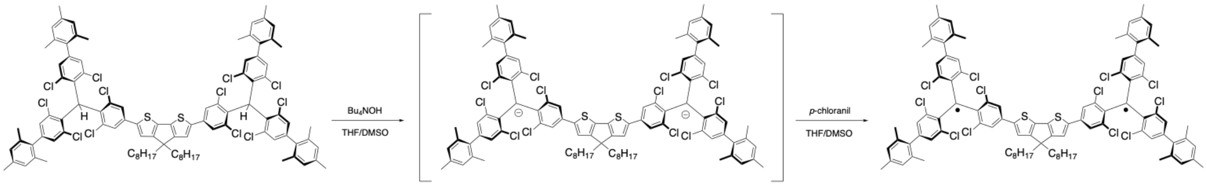

To a flame-dried Schlenk tube was added $(\alpha HM_2TTM)_2$–CDT (268 mg, 0.268 mmol, 1.00 equiv.), THF (25.6 mL, 0.0105 M) and DMSO (76.7 mL, 0.00350 M), and the solution was purged with a continuous stream of Ar for 0.5 h. To the degassed solution was then added 40% aqueous ammonium hydroxide (1.57 mL, 2.42 mmol, 9.00 equiv., purged with a continuous stream of Ar for 0.5 h prior to addition) in the absence of light. After stirring for 12 h at room temperature under an atmosphere of Ar, *p*-chloranil (660 mg, 2.68 mmol, 10.00 equiv.) was added and the mixture was further stirred for 1 h at room temperature. The mixture was added to methanol dropwise and the precipitates were collected by vacuum filtration. The crude product was purified with column chromatography, by gradually increasing the eluent polarity from hexane to 4% (v/v) EtOAc in hexane. The target compound was collected as a deep purple solid (206 mg, 77% yield). TOF-MS-ES^+ Calcd. $[C_{99}H_{92}Cl_{12}S_2]^-$: 1765.2981. Found: m/z = 1765.2960. EA Calcd. for $C_{99}H_{92}Cl_{12}S_2$: C, 67.13; H, 5.24. Found: C, 67.09; H, 5.29.

Synthesis of p(M_1TTM–CDT) polyradical via radical conversion

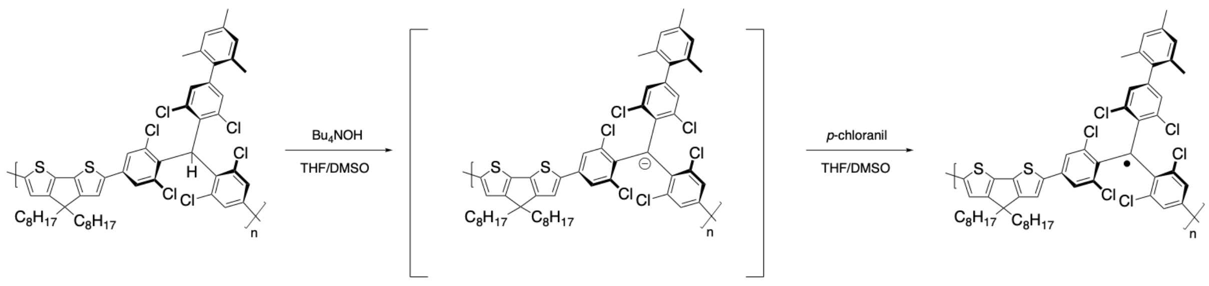

To a flame-dried Schlenk tube was added αHM_1TTM–CDT (150 mg, 0.150 mmol, 1.00 equiv.), THF (14.3 mL, 0.0105 M) and DMSO (42.9 mL, 0.00350 M), and the solution was purged with a continuous stream of Ar for 0.5 h. To the degassed solution was then added 40% aqueous ammonium hydroxide (0.390 mL, 0.601 mmol, 4.00 equiv. per monomer unit, purged with a continuous stream of Ar for 0.5 h prior to addition) in the absence of light. After stirring for 72 h at room temperature under an atmosphere of Ar, *p*-chloranil (370 mg, 1.50 mmol, 10.00 equiv. per monomer unit) was added and the mixture was further stirred for 1 h at room temperature. The mixture was added to methanol dropwise and the precipitates were collected by vacuum filtration. The dark solids were subjected to three cycles of Soxhlet extraction using methanol, hexane, and acetone as solvents, and the polymer was eventually collected from the chloroform fraction. The chloroform fraction was concentrated *in vacuo* and slowly added to methanol, the precipitates were collected via vacuum filtration to afford the target polymer as a deep purple solid (131 mg, 88% yield). GPC: M_n = 8.6 kg/mol, M_w = 13 kg/mol, $Đ$ = 1.48. EA Calcd. for $C_{55}H_{59}Cl_6S_2$: C, 66.21; H, 6.00. Found: C, 67.09; H, 5.29.

Synthesis of M_2TTM–CDT–Ph monoradical via radical conversion

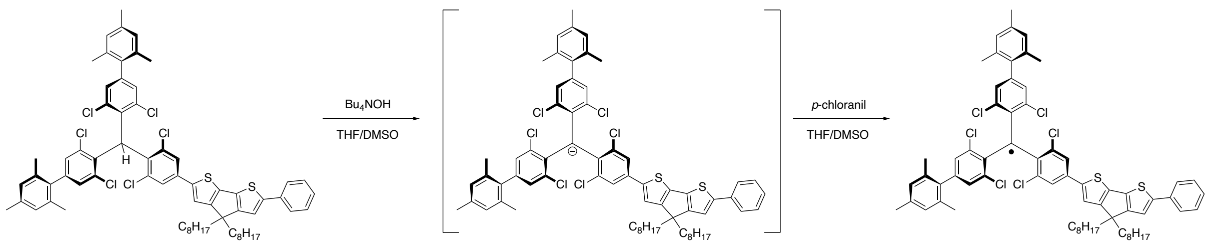

To a flame-dried Schlenk tube was added αHM_2TTM–CDT (150 mg, 0.150 mmol, 1.00 equiv.), THF (14.3 mL, 0.0105 M) and DMSO (42.9 mL, 0.00350 M), and the solution was purged with a continuous stream of Ar for 0.5 h. To the degassed solution was then added 40% aqueous ammonium hydroxide (0.390 mL, 0.601 mmol, 4.00 equiv., purged with a continuous stream of Ar for 0.5 h prior to addition) in the absence of light. After stirring for 12 h at room temperature under an atmosphere of Ar, *p*-chloranil (367 mg, 1.50 mmol, 10.00 equiv.) was added and the mixture was further stirred for 1 h at room temperature. The mixture was added to methanol dropwise and the precipitates were collected by vacuum filtration. The crude product was purified with column chromatography, by gradually increasing the eluent polarity from hexane to 4% (v/v) EtOAc in hexane. The target compound was collected as a deep purple solid (118 mg, 79% yield). TOF-MS-ASAP$^-$ Calcd. $[C_{68}H_{69}Cl_6S_2]^-$: 1159.2972. Found: *m/z* = 1159.2930. EA Calcd. for $C_{68}H_{69}Cl_6S_2$: C, 70.22; H, 5.98. Found: C, 69.94; H, 5.84.

Physicochemical Properties

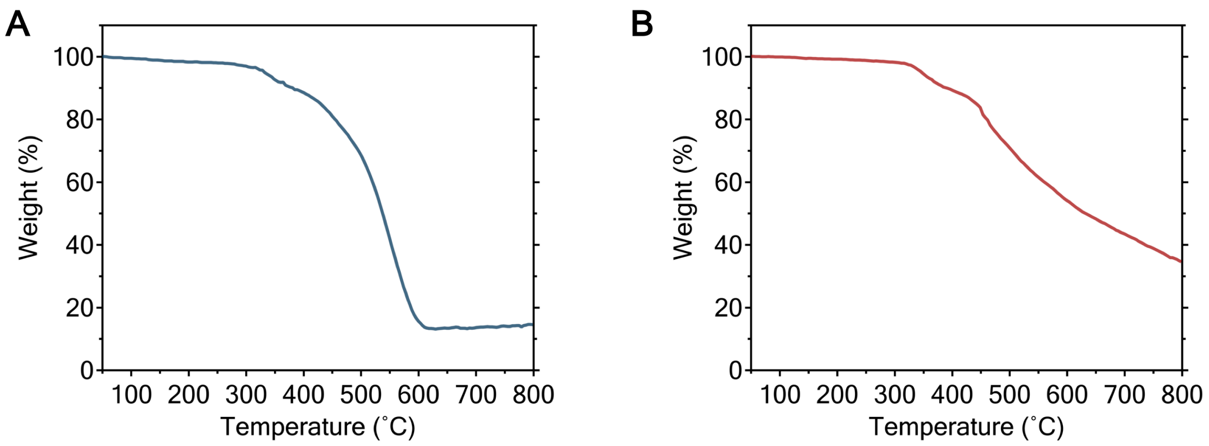

Fig. S3.

Thermal stability of radicals. TGA curves of (**A**) $(M_2TTM)_2$–CDT and (**B**) p(M_1TTM–CDT).

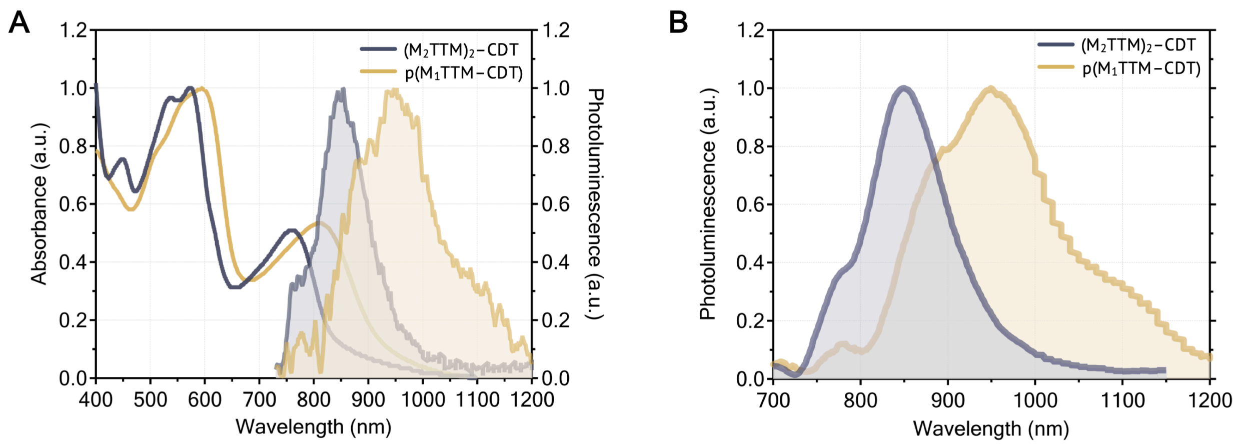

Fig. S4.

Photophysical properties. (**A**) Film-state absorption and emission spectra of $(M_2TTM)_2$–CDT and p(M_1TTM–CDT). (**B**) Emission profiles for diluted films with 5 wt% of radicals in a polystyrene matrix.

Transient Absorption Spectroscopy

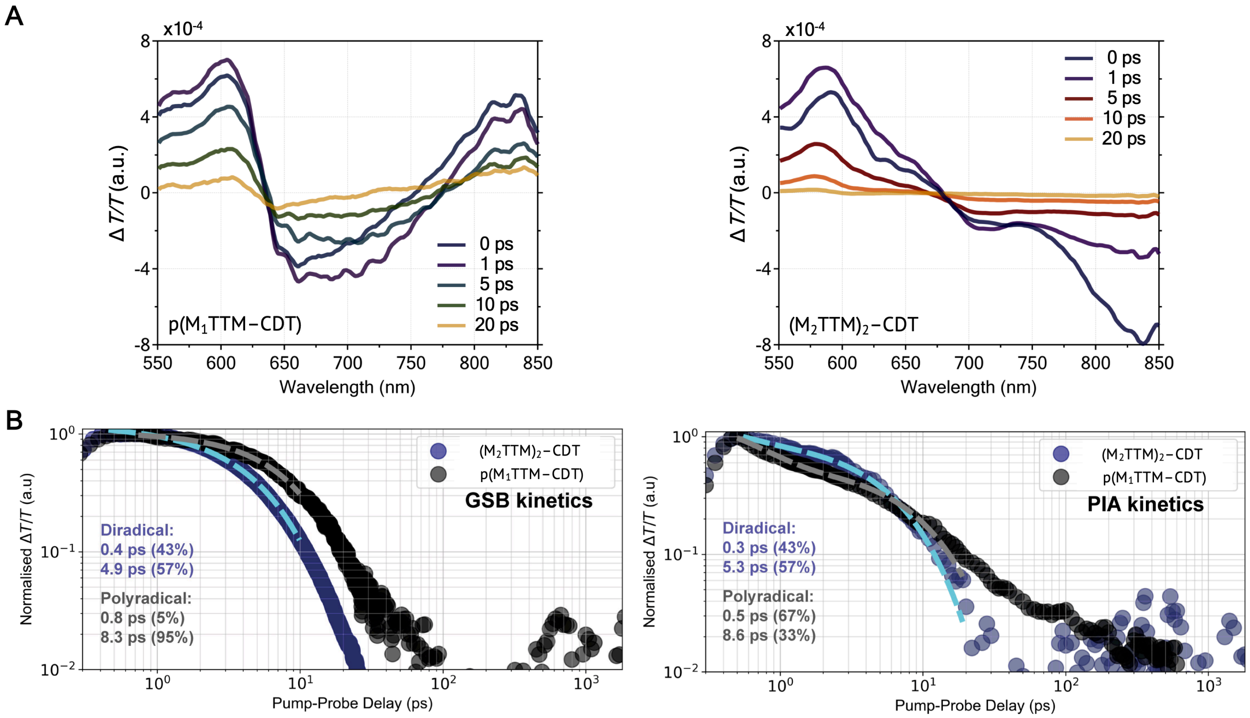

Fig. S5.

Femtosecond transient absorption spectroscopy. (**A**) Transient absorption spectra of $(M_2TTM)_2$–CDT and p(M_1TTM–CDT) thin films. (**B**) Kinetics of ground-state bleach and photo-induced absorption of $(M_2TTM)_2$–CDT and p(M_1TTM–CDT).

Quantum Chemistry Calculations

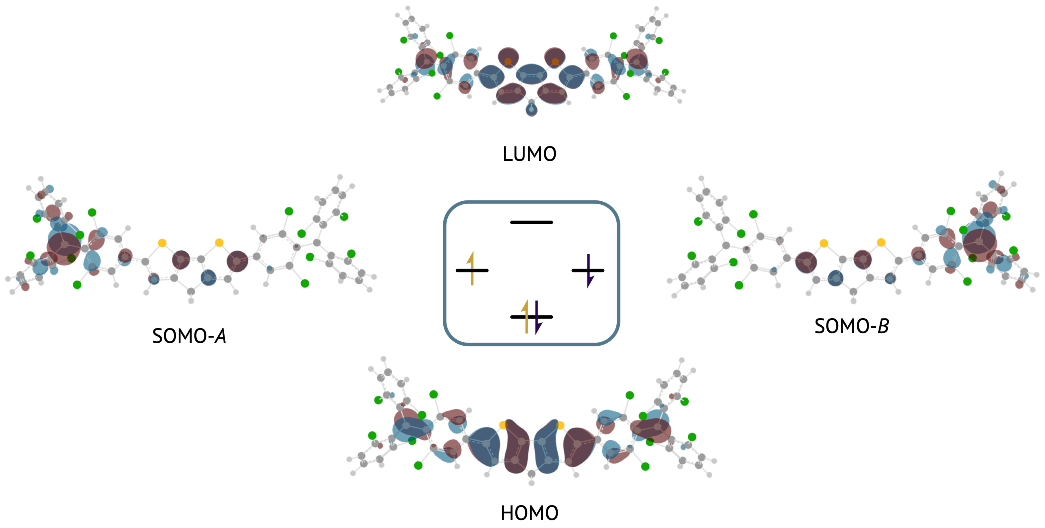

Fig. S6.

Computational analysis of excited states. Depiction of frontier molecular orbitals of $(M_2TTM)_2$–CDT computed as state-averaged CASSCF natural orbitals. The SOMOs were localized on the radical centers in order to facilitate the subsequent analysis in terms of diradical and zwitterionic states.

Table S1. Detailed calculation on the electronic transitions. Vertical excitation energies (ΔE in eV), oscillator strengths (f) and character assignment of the lowest lying singlet and triplet excited states of $(M_2TTM)_2$–CDT computed at the MS-CASPT2(6,6) level of theory.

State	ΔE	f	Character
S_0 (^{1}A')	0.000		$SOMO^2$ (dirad, 73%)
T_1 (^{3}A'')	0.008		$SOMO^2$ (dirad, 90%)
S_1 (^{1}A'')	1.347	0.275	$SOMO^2$ (ionic, 50%), H→S (25%)
T_2 (^{3}A')	1.783		H→S (35%), H→L (19%), S→L (18%)
S_2 (^{1}A')	1.813	0.000	H→L (25%), H→S (17%), S→L (14%)
S_3 (^{1}A'')	2.217	0.058	S→L (28%), H_1→S (20%), SL_1 (12%)
T_3 (^{3}A'')	2.283		S→L (75%)

Table S2. Analysis of the wave functions obtained. Single-excitation character (Ω), exciton size (d_{exc}), number of unpaired electrons ($n_{u,nl}$), and promotion number p, computed at the MS-CASPT2 level.

State	Ω	d_{exc} (Å)	$n_{u,nl}$	p
S_0 (^{1}A’)			2.120	
T_1 (^{3}A”)	0.985	2.727	2.098	0.329
S_1 (^{1}A”)	0.357	9.632	2.276	1.927
T_2 (^{3}A’)	0.102	6.764	3.540	1.154
S_2 (^{1}A’)	0.094	7.200	3.447	1.001
S_3 (^{1}A”)	0.206	8.067	2.967	1.619
T_3 (^{3}A”)	0.912	3.629	4.013	1.092

A detailed analysis of the wave functions was performed to obtain more insight into excited state characters (*62*). We note the significantly enhanced exciton size (d_{exc} = 9.6 Å) of the S_1 state representing its charge separated (that is zwitterionic) character.

Table S3. Analysis of solvatochromic shifts. Vertical excitation energies for non-equilibrium [ΔE (non-eq.)] and equilibrium solvation [ΔE (eq.)] computed in acetonitrile (MeCN) and dimethylsulfoxide (DMSO) for $(M_2TTM)_2$–CDT at the MS-CASPT2(6,6) level of theory.

State	Solvent	ΔE (non-eq.)	ΔE (eq.)
S_1 (^{1}A”)	MeCN	1.184	1.046
S_1 (^{1}A”)	DMSO	1.168	1.048

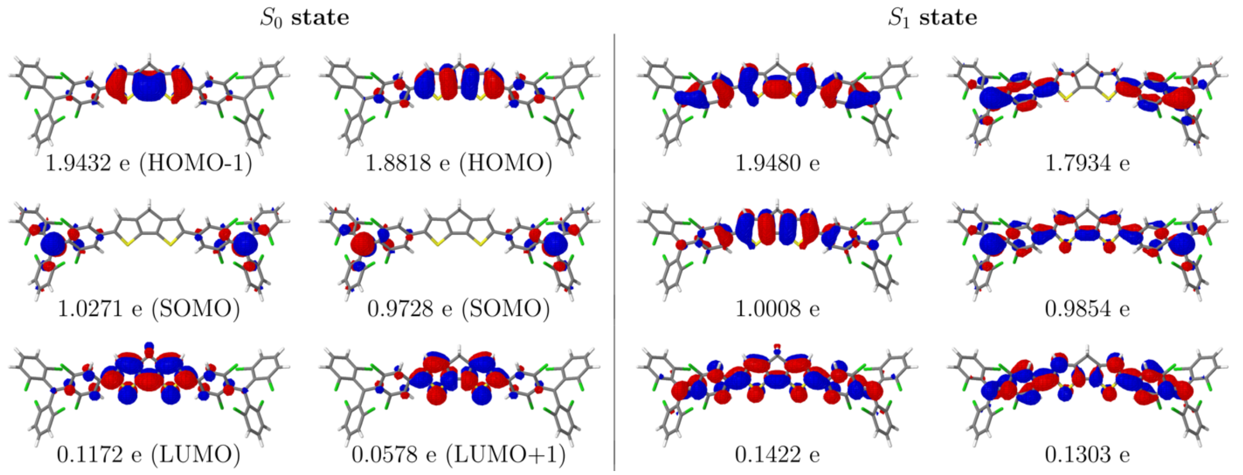

Fig. S7.

Analysis of the S_0 and S_1 states of $(TTM)_2$-CDT in terms of their state-specific natural orbitals computed at the MS-CASPT2 level. Occupation numbers are given below each plot. This figure illustrates that in the S_0 state the unpaired electrons are quite localized on the TTM units with only secondary contributions on the bridge. Conversely, enhanced delocalization is seen for S_1. The orbital space spanned by these six orbitals represents the CAS(6,6) active space used in the computations.

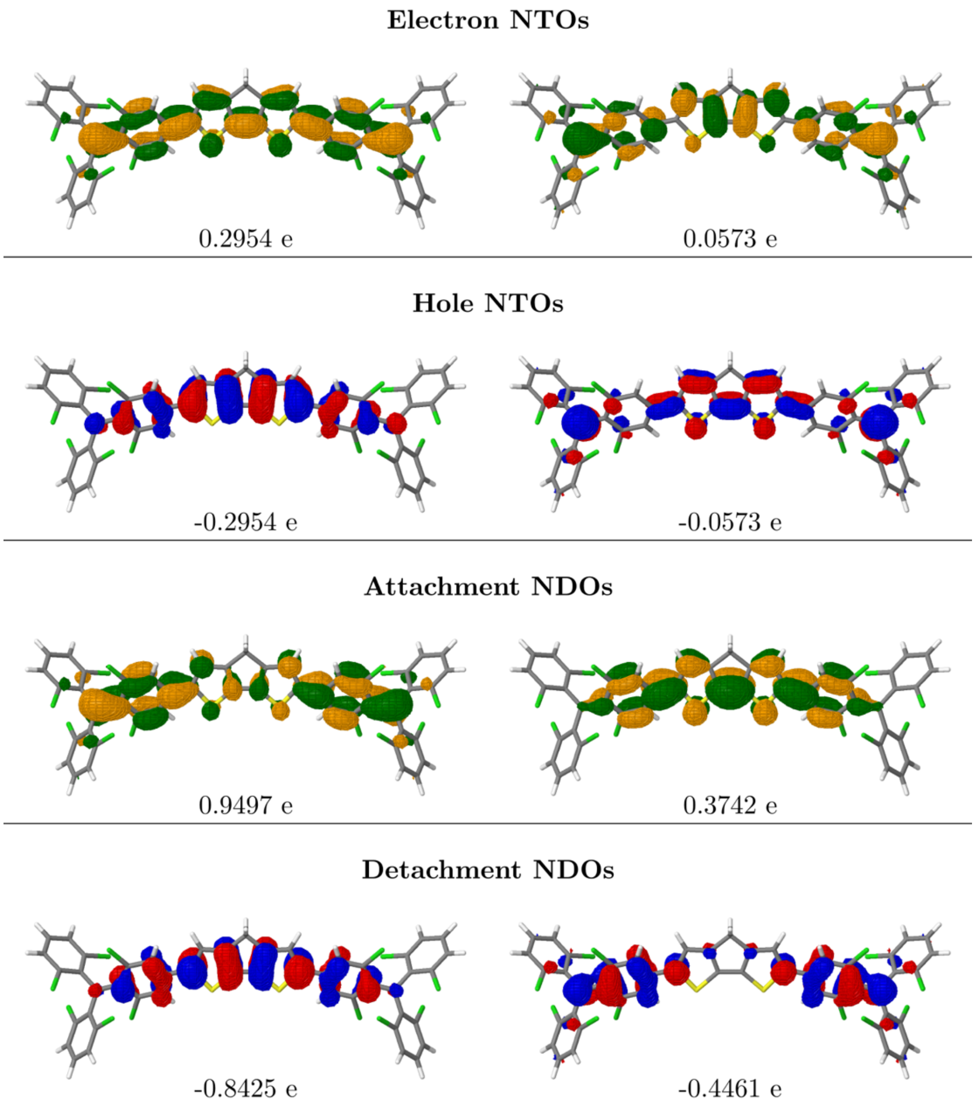

Fig. S8.

Analysis of the $S_0 \rightarrow S_1$ electron transition in terms of natural transition orbitals (NTOs) and natural difference orbitals (NDOs) as computed from MS-CASPT2. Associated eigenvalues are shown below. Both representations reflect the delocalized nature of the excitation. The difference between NTOs and NDOs derives from partial doubly excited character of the excitation.

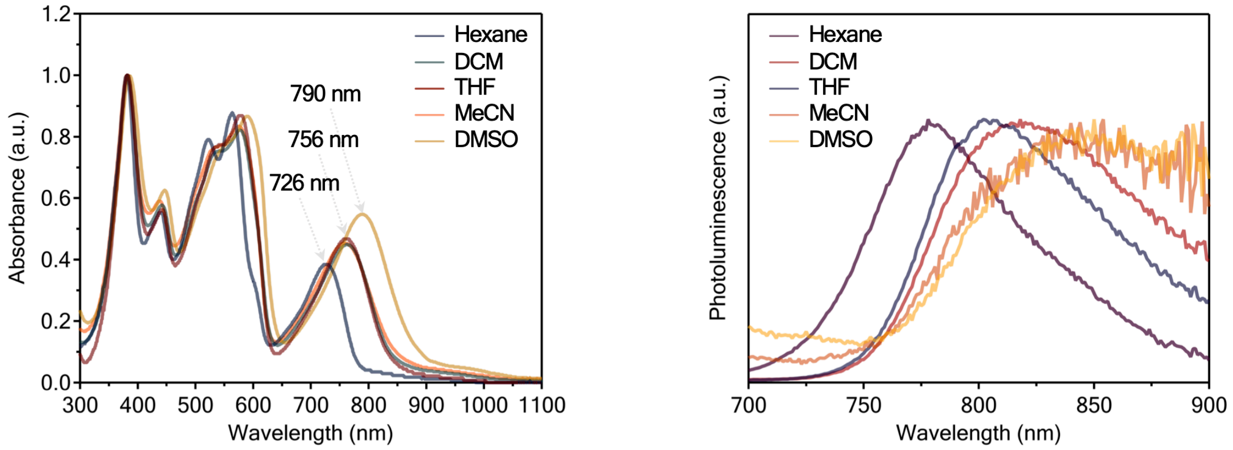

Fig. S9.

Solvatochromism of $(M_2TTM)_2$–CDT. Solvent dependence of UV-vis-NIR absorption (left) and photoluminescence (right) demonstrating the polar nature of the zwitterionic excited state.

Electron Paramagnetic Resonance Spectroscopy (EPR)

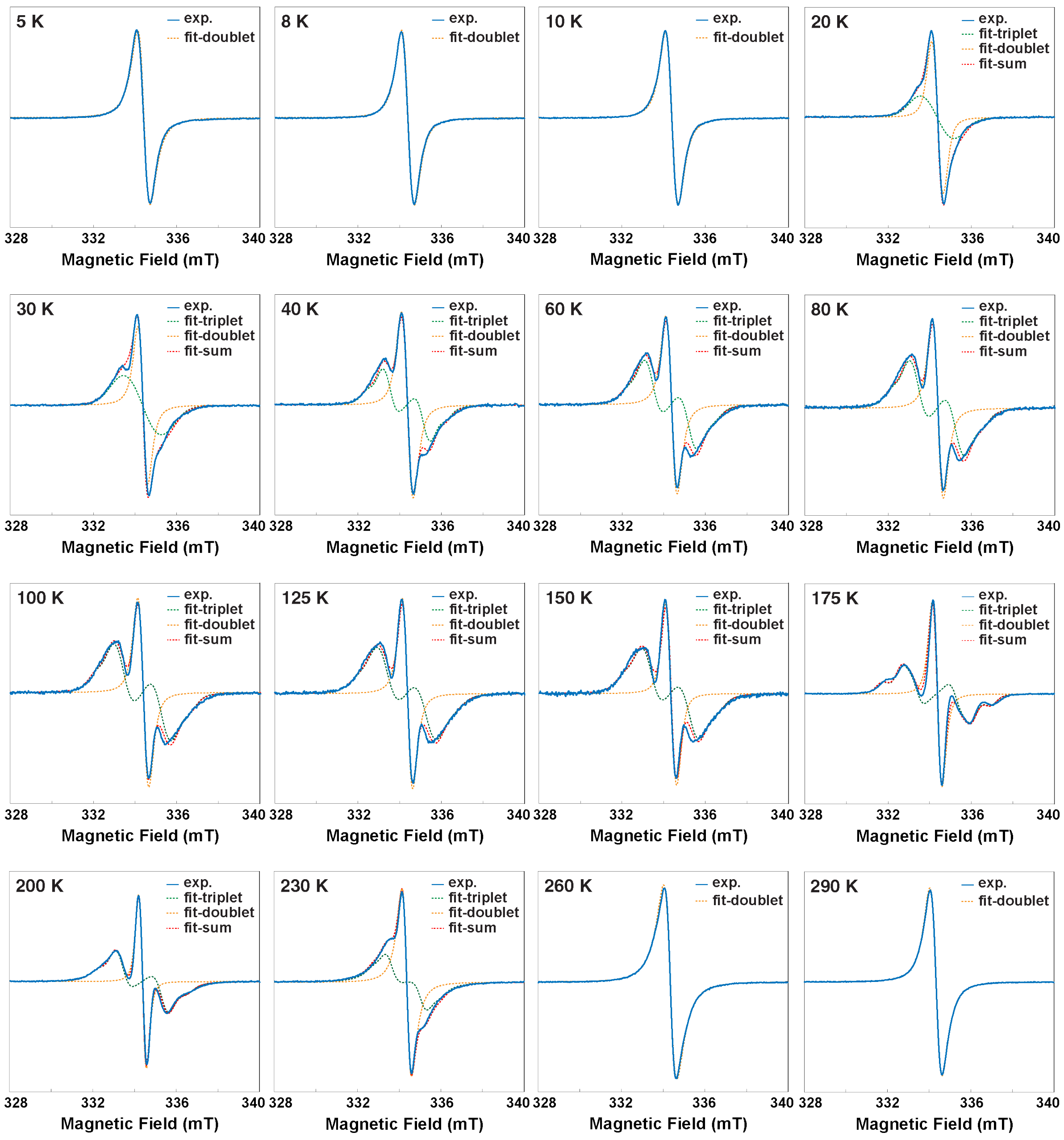

Fig. S10.

Temperature-dependent EPR Spectra of $(M_2TTM)_2$–CDT. Experimental (blue) and fitted (yellow) EPR spectra from 5 to 290 K in toluene solution (50 µM). The fitting parameters are listed in Table S4. All spectra are adjusted to a similar intensity.

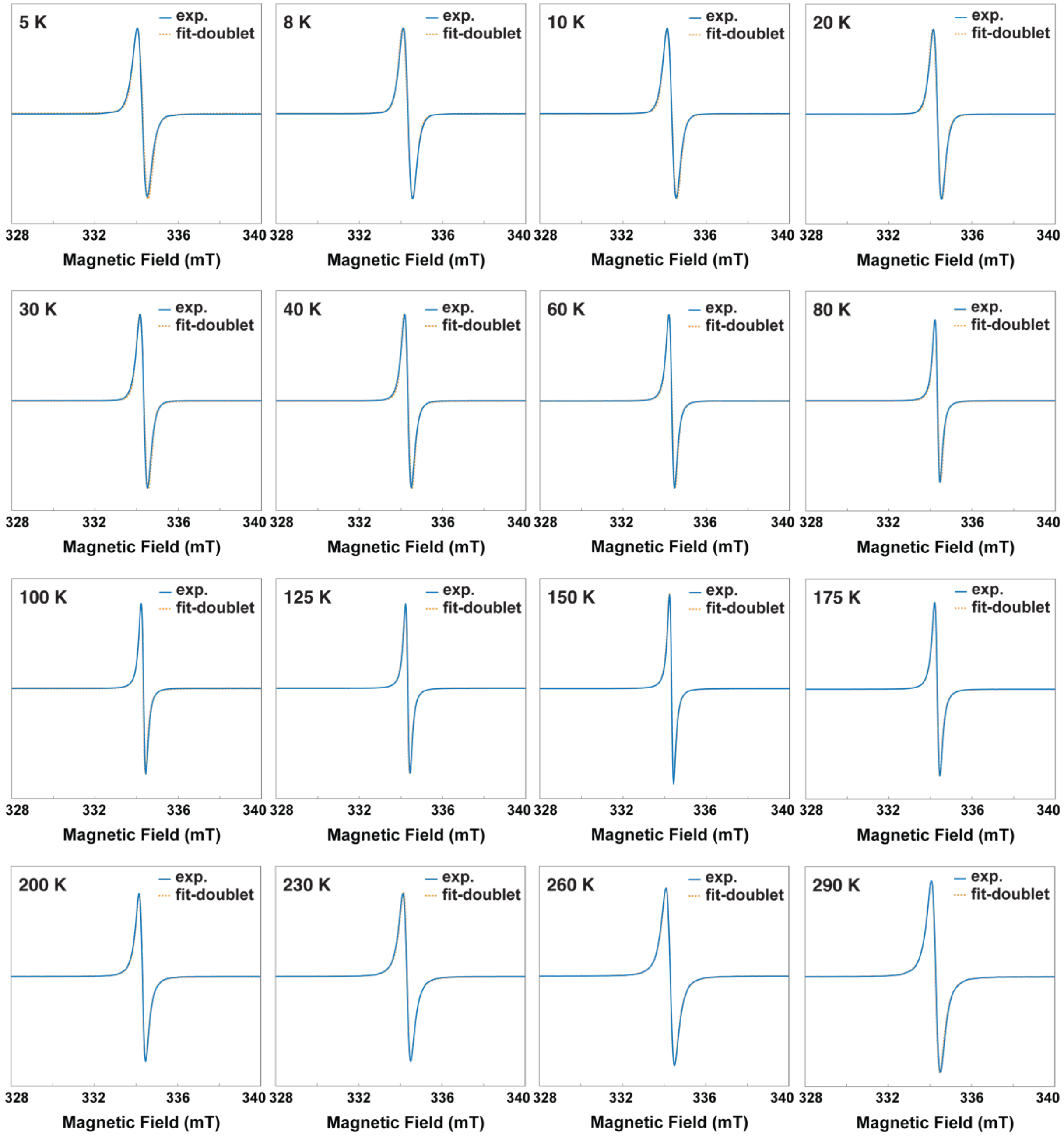

Fig. S11.

Temperature-dependent EPR Spectra of p(M_1TTM–CDT). Experimental (blue) and fitted (yellow) EPR spectra from 5 to 290 K in toluene solution (50 μM). The fitting parameters are listed in Table S5. All spectra are adjusted to a similar intensity.

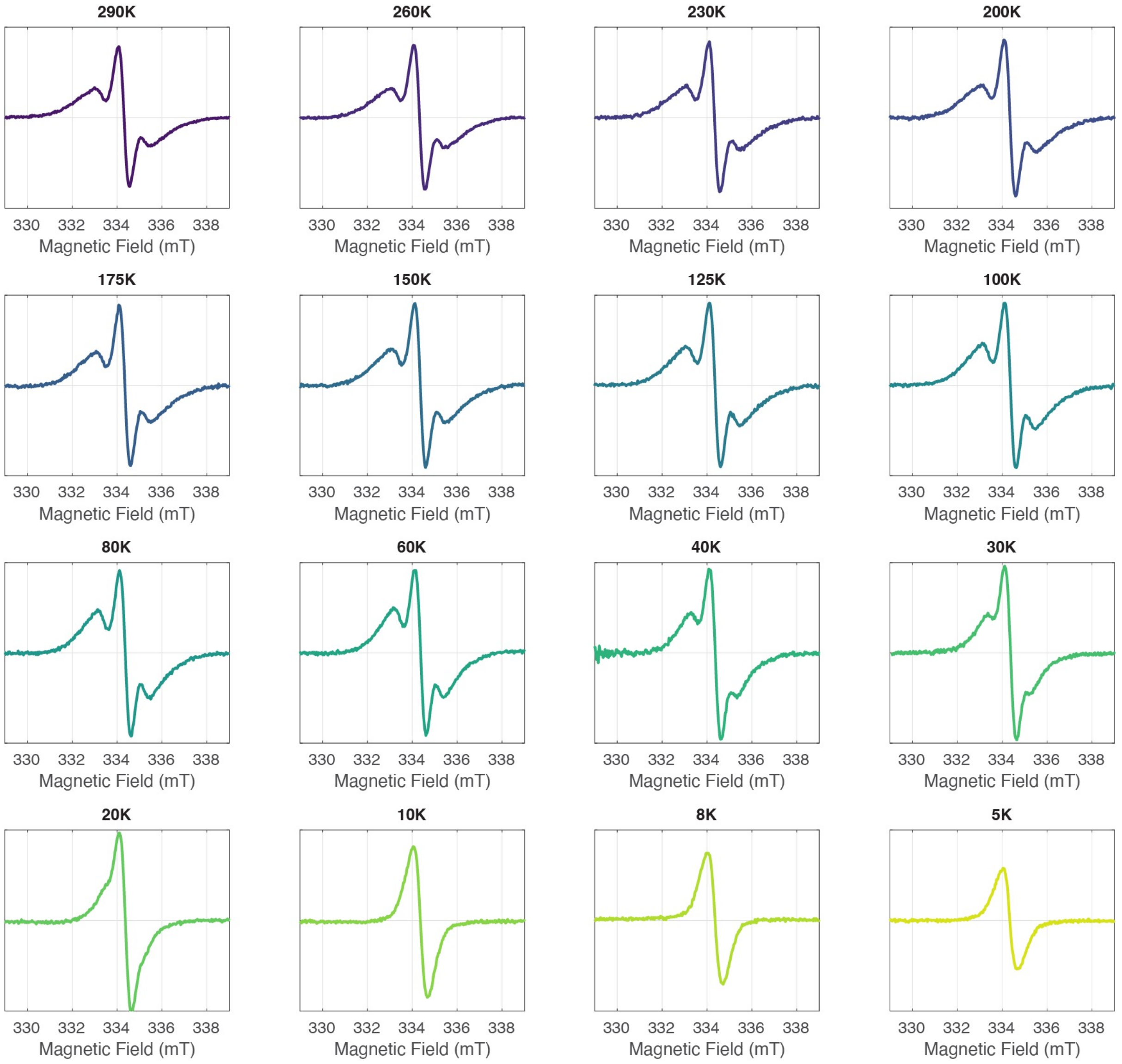

Fig. S12.

Temperature-dependent EPR Spectra of (M_2TTM)$_2$–CDT in the film state. Experimental EPR spectra from 5 to 290 K in the film state, which was diluted with PMMA at a concentration of 1 wt%. The fitting parameters are listed in Table S6.

Table S4. Fitting parameter for various-temperature EPR spectra on the $(M_2TTM)_2$–CDT sample. All spectra were fitted using the Voigt line shape, which incorporates both Gaussian and Lorentzian functions. The zero-field splitting (ZFS) parameters, encompassing the D tensor and E tensor, are detailed within the triplet state part.

T(K)	Triplet state part						Doublet state part		
	g tensor	peak-to-peak linewidth (Gaussian, mT)	peak-to-peak linewidth (Lorentzian, mT)	ZFS, D (MHz)	Estimated spin-spin distance (nm)	ZFS, E (MHz)	g tensor	peak-to-peak linewidth (Gaussian, mT)	peak-to-peak linewidth (Lorentzian, mT)
290	/	/	/	/	/	/	2.0027	0.28	0.87
260	/	/	/	/	/	/	2.0027	0.03	1.10
230	2.0029	0.14	1.04	22.7	1.32	22.7	2.0027	0.12	0.76
200	2.0029	0.42	0.69	25.3	1.27	32.0	2.0027	0.33	0.29
175	2.0029	0.52	0.32	26.1	1.26	40.1	2.0027	0.39	0.39
150	2.0029	0.68	0.55	23.6	1.31	34.0	2.0027	0.46	0.40
125	2.0029	0.95	0.43	34.0	1.15	30.9	2.0027	0.36	0.54
100	2.0029	0.68	0.45	23.0	1.31	34.0	2.0027	0.40	0.53
80	2.0029	0.63	0.49	23.1	1.31	32.7	2.0027	0.36	0.60
60	2.0029	0.73	0.45	32.5	1.17	27.6	2.0027	0.38	0.60
40	2.0029	0.44	0.51	32.6	1.17	24.5	2.0027	0.43	0.57
30	2.0029	1.20	0.41	30.5	1.19	10.2	2.0027	0.31	0.58
20	2.0029	0.89	0.55	28.6	1.22	7.5	2.0027	0.47	0.43
10	/	/	/	/	/	/	2.0027	0.38	0.77
8	/	/	/	/	/	/	2.0027	0.35	0.81
5	/	/	/	/	/	/	2.0027	0.21	0.94

Table S5. Fitting parameter for various-temperature EPR spectra on the p(M_1TTM–CDT) sample. Above 100 K, the spectra were fitted using the pure Lorentzian function. Conversely, below 100 K, the spectra were fitted using Voigt lineshapes, which combine both Gaussian and Lorentzian functions.

T(K)	g tensor (doublet)	peak-to-peak linewidth (Gaussian, mT)	peak-to-peak linewidth (Lorentzian, mT)
290	2.0028	/	0.70
260	2.0028	/	0.67
230	2.0027	/	0.62
200	2.0027	/	0.52
175	2.0028	/	0.41
150	2.0028	/	0.35
125	2.0028	/	0.36
100	2.0028	/	0.35
80	2.0026	0.18	0.30
60	2.0026	0.24	0.30
40	2.0027	0.33	0.27
30	2.0026	0.39	0.24
20	2.0027	0.44	0.22
10	2.0027	0.48	0.23
8	2.0028	0.48	0.25
5	2.0029	0.47	0.36

Table S6. Fitting parameter for various-temperature EPR spectra on the $(M_2TTM)_2$–CDT sample in the film state. The film was diluted with PMMA at a concentration of 1 wt%. All the spectra were fitted using the pure Lorentzian function.

T (K)	Triplet state part				Doublet state part	
	g tensor	peak-to-peak linewidth (Lorentzian, mT)	ZFS, D (MHz)	Estimated spin-spin distatance (nm)	g tensor	peak-to-peak linewidth (Lorentzian, mT)
290	2.0047	0.7	68.9	0.91	2.0045	0.5
260	2.0047	0.71	68.8	0.91	2.0045	0.5
230	2.0047	0.71	68.6	0.91	2.0045	0.5
200	2.0047	0.7	68.1	0.91	2.0044	0.47
175	2.0047	0.67	67.5	0.92	2.0044	0.5
150	2.0046	0.69	67.2	0.92	2.0043	0.5
125	2.0047	0.72	66.9	0.92	2.0044	0.5
100	2.0046	0.68	64.9	0.93	2.0044	0.5
80	2.0042	0.71	62	0.94	2.0043	0.47
60	2.0042	0.59	60.3	0.95	2.0044	0.51
40	2.0044	0.57	53.5	0.99	2.0044	0.52
30	2.0047	0.93	43.4	1.06	2.0044	0.47
20	2.0045	0.65	33.3	1.16	2.0043	0.52
10					2.0045	0.64
8					2.0045	0.65
5					2.0045	0.69

Superconducting Quantum Interference Device (SQUID)

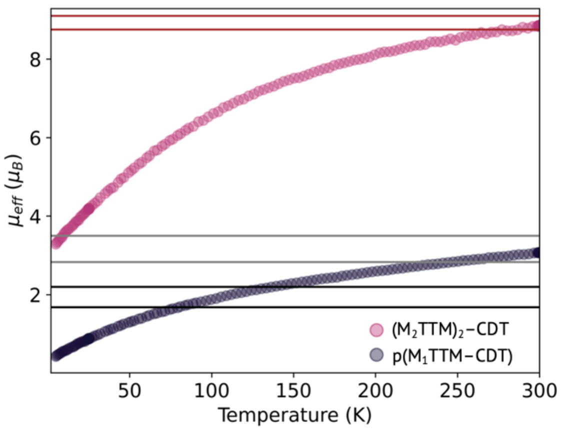

Fig. S13.

Investigation of the unpaired electrons using SQUID. Temperature-dependent μ_{eff} of $(M_2TTM)_2$–CDT and p(M_1TTM–CDT) in the solid-state.

Cyclic Voltammetry

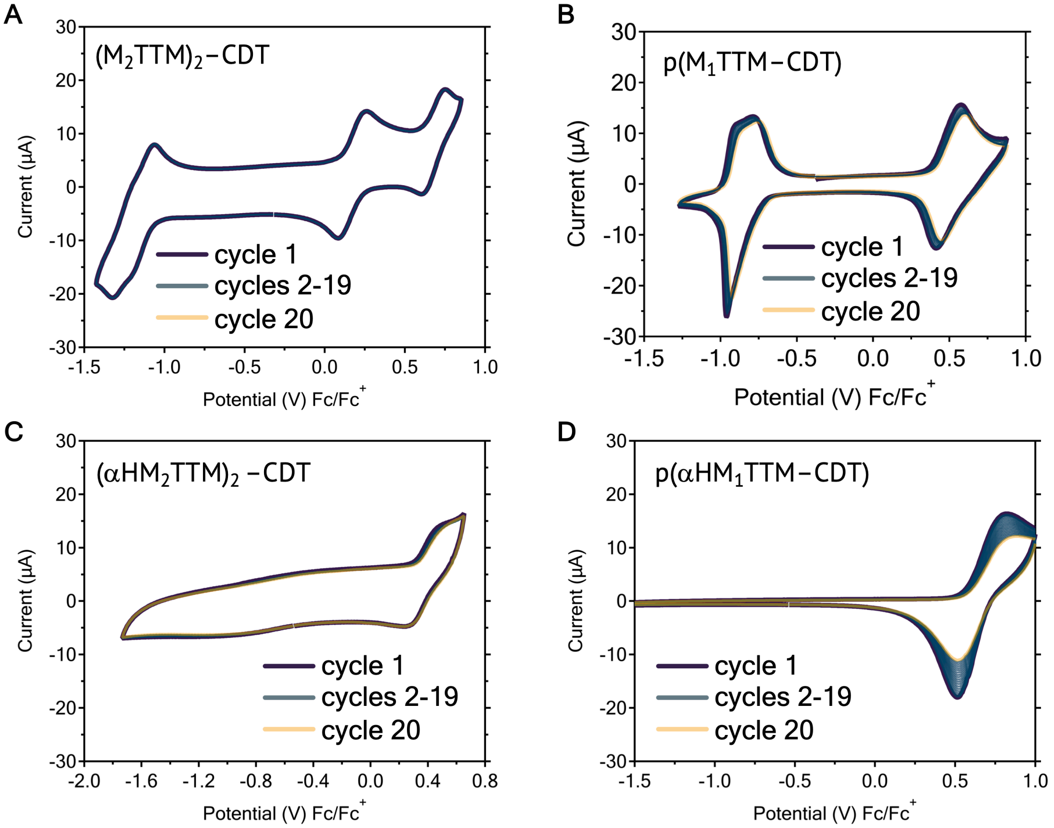

Fig. S14.

Electrochemical properties of radicals and their corresponding precursors. Cyclic voltammograms of (**A**) $(M_2TTM)_2$–CDT, (**B**) p(M_1TTM–CDT), (**C**) $(\alpha HM_2TTM)_2$–CDT, and (**D**) p(αHM_1TTM–CDT).

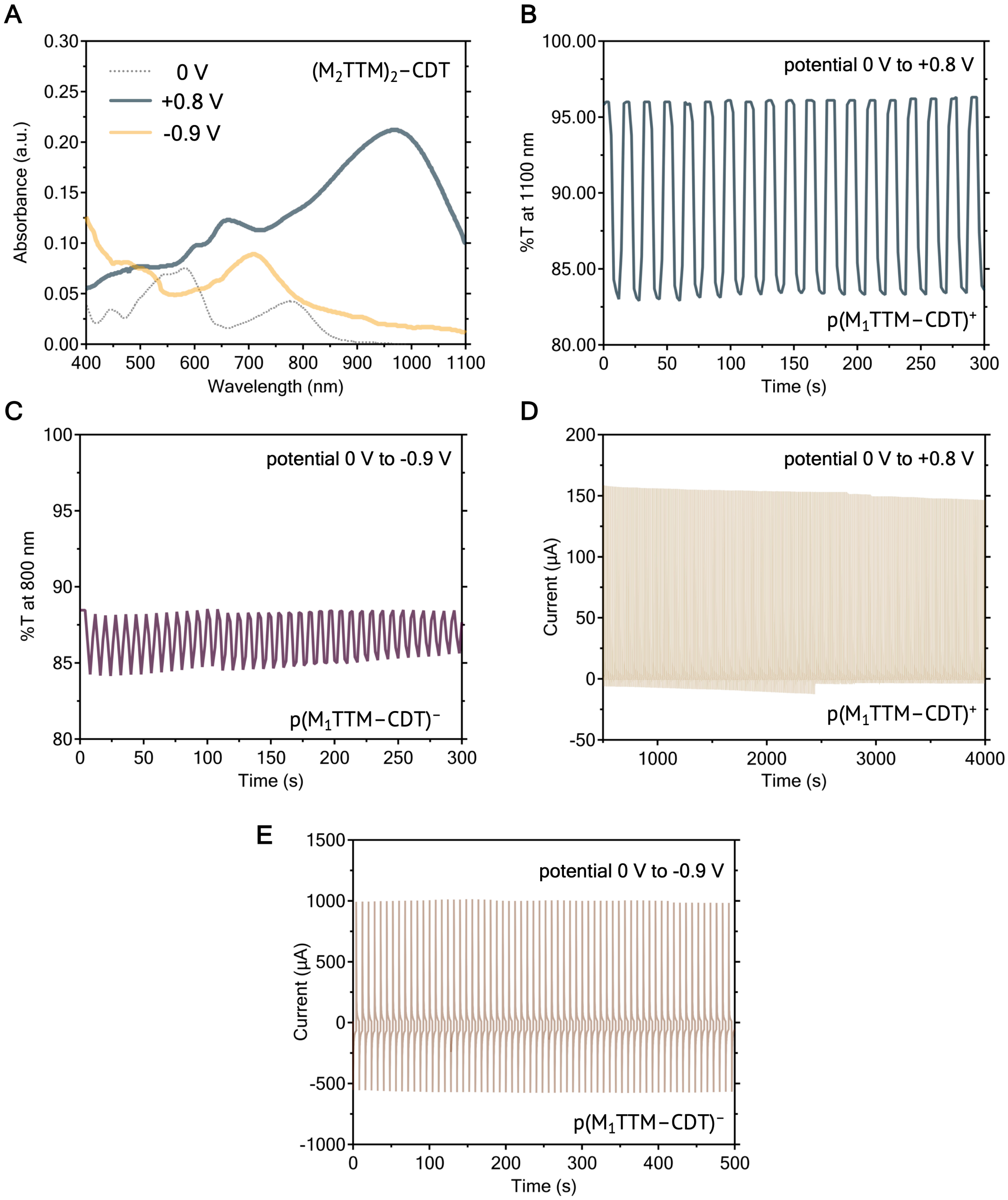

Fig. S15.

Electrochromic properties of radicals. (**A**) Film-state (spin-coated on ITO glass substrates) spectroelectrochemical spectra of $(M_2TTM)_2$–CDT at its neutral, oxidized, and reduced states, (**B**) Transmittance monitored at 1100 nm during the oxidation cycles, (**C**) Transmittance monitored at 800 nm during the reduction cycles, and (**D–E**) Full oxidation and reduction cycles of p(M_1TTM–CDT) thin film.

NMR Spectra

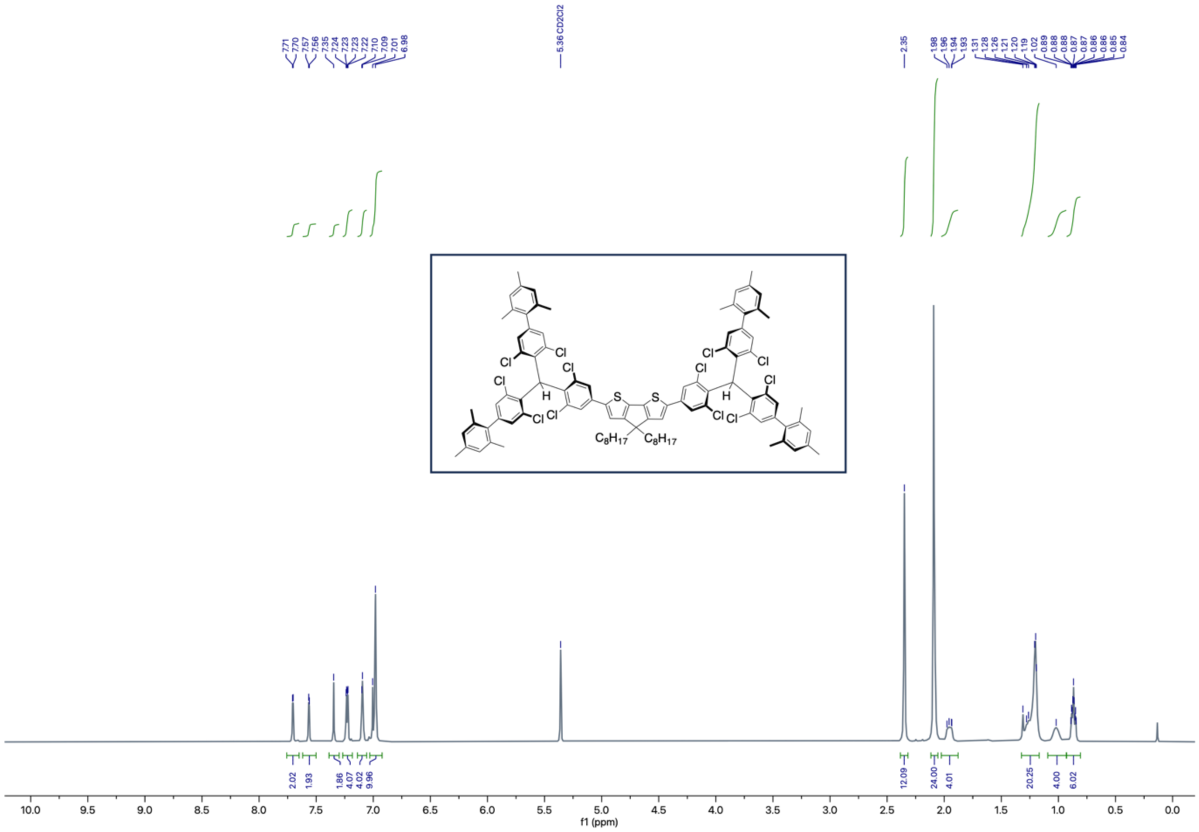

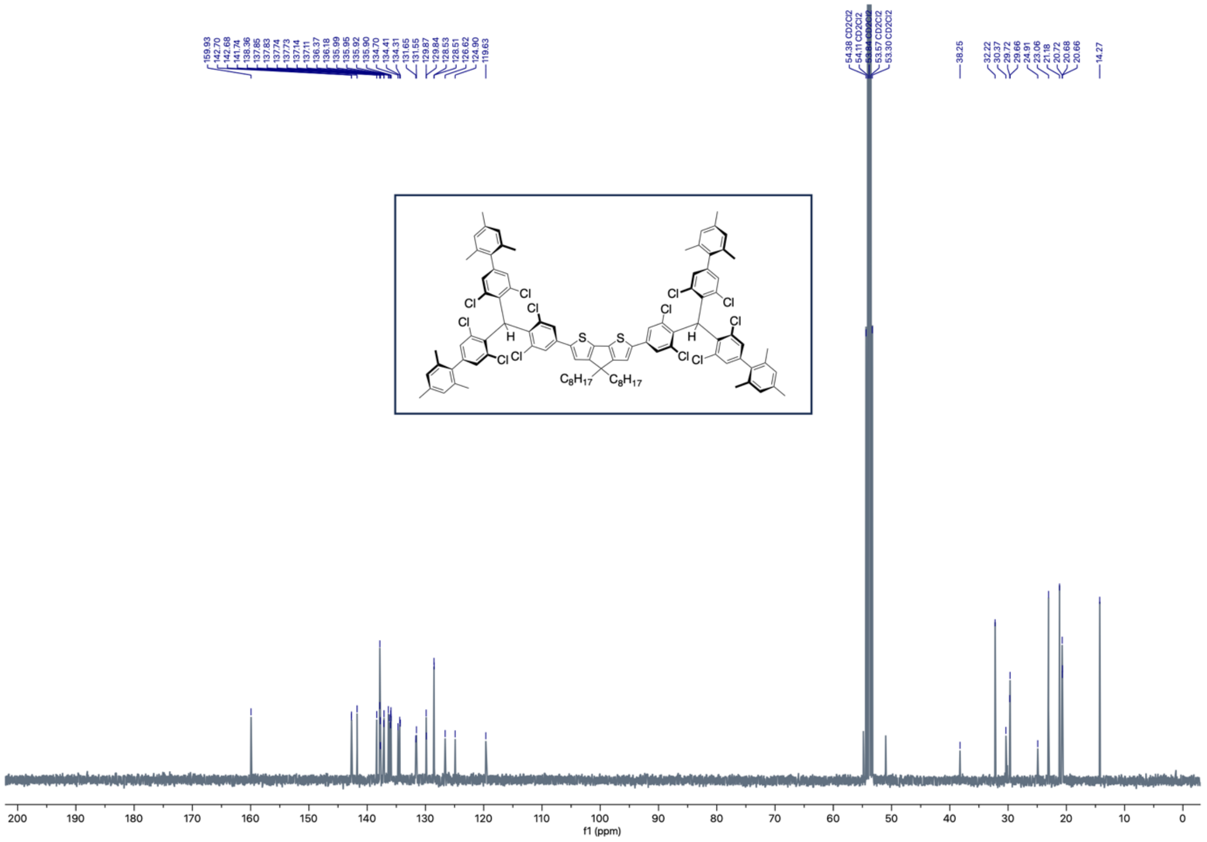

159.93
142.70
142.68
141.74
138.36
137.85
137.83
137.74
137.73
137.14
137.11
136.37
136.18
135.99
135.95
135.92
135.90
134.70
134.41
134.31
131.65
131.55
129.87
129.84
128.53
128.51
126.62
124.90
119.63
54.38 CD2Cl2
54.11 CD2Cl2
53.57 CD2Cl2
53.30 CD2Cl2
38.25
32.22
30.37
29.72
29.66
24.91
23.06
21.18
20.72
20.68
20.66
14.27
Cl
H
S
C_8H_{17}
200
190
180
170
160
150
140
130
120
110
100
90
80
70
60
50
40
30
20
10
0
f1 (ppm)

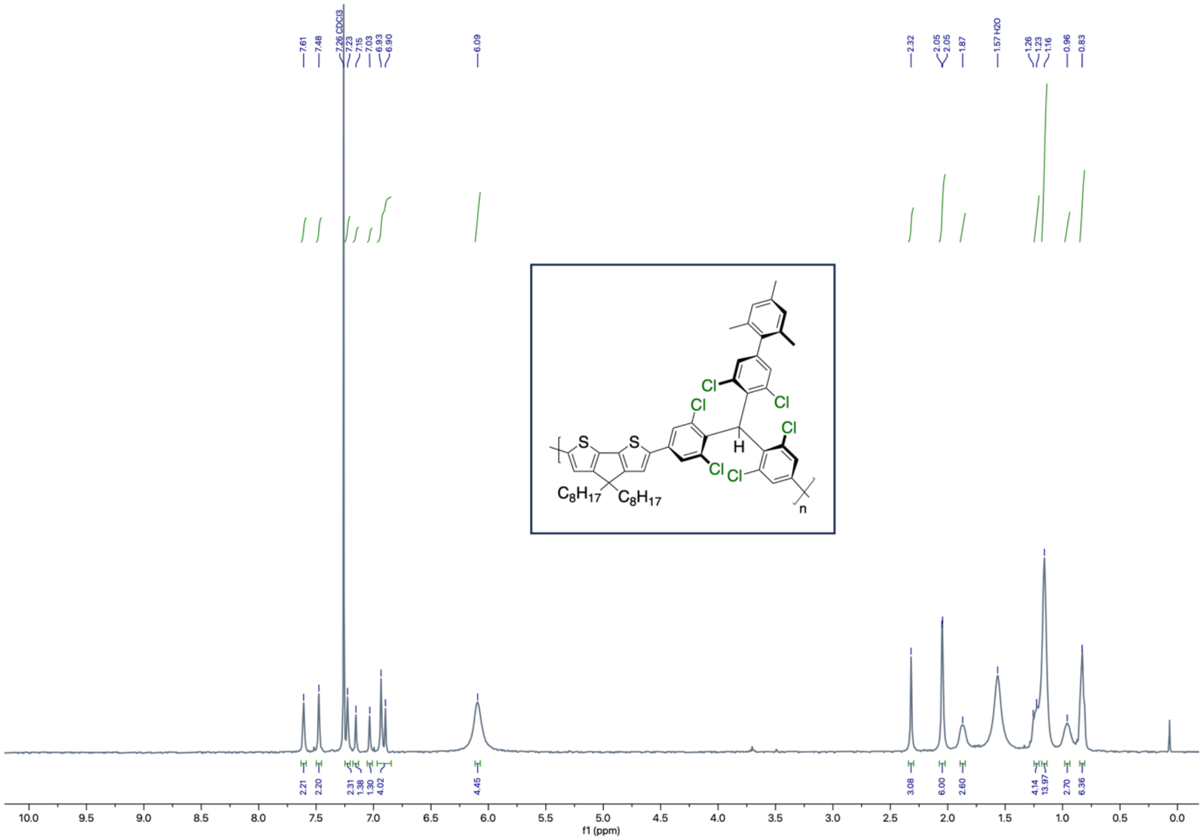

7.61
7.48
7.26 CDCl3
7.23
7.15
7.03
6.93
6.90
6.09
2.32
2.05
2.05
1.87
1.57 H2O
1.26
1.23
1.16
0.96
0.83
S
S
Cl
Cl
Cl
Cl
Cl
Cl
H
C8H17
C8H17
n
2.21
2.20
2.31
1.38
1.30
4.02
4.45
3.08
6.00
2.60
4.14
13.97
2.70
6.36
10.0
9.5
9.0
8.5
8.0
7.5
7.0
6.5
6.0
5.5
5.0
4.5
4.0
3.5
3.0
2.5
2.0
1.5
1.0
0.5
0.0
f1 (ppm)

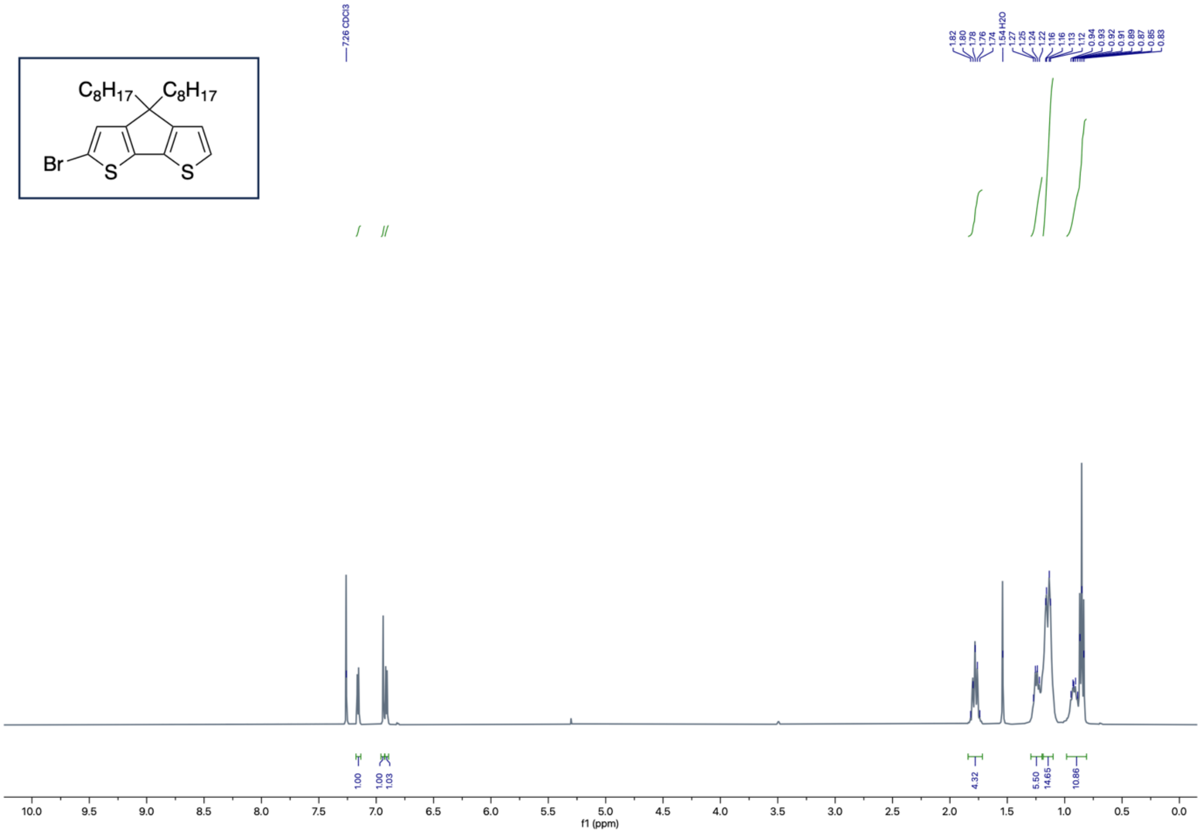

C8H17 C8H17
Br
S
S
7.26 CDCl3
1.82
1.80
1.78
1.76
1.74
1.54 H2O
1.27
1.25
1.24
1.22
1.16
1.16
1.13
1.13
1.12
0.94
0.93
0.92
0.91
0.89
0.87
0.85
0.83
1.00
1.00
1.03
4.32
5.50
14.65
10.86
10.0 9.5 9.0 8.5 8.0 7.5 7.0 6.5 6.0 5.5 5.0 4.5 4.0 3.5 3.0 2.5 2.0 1.5 1.0 0.5 0.0
f1 (ppm)

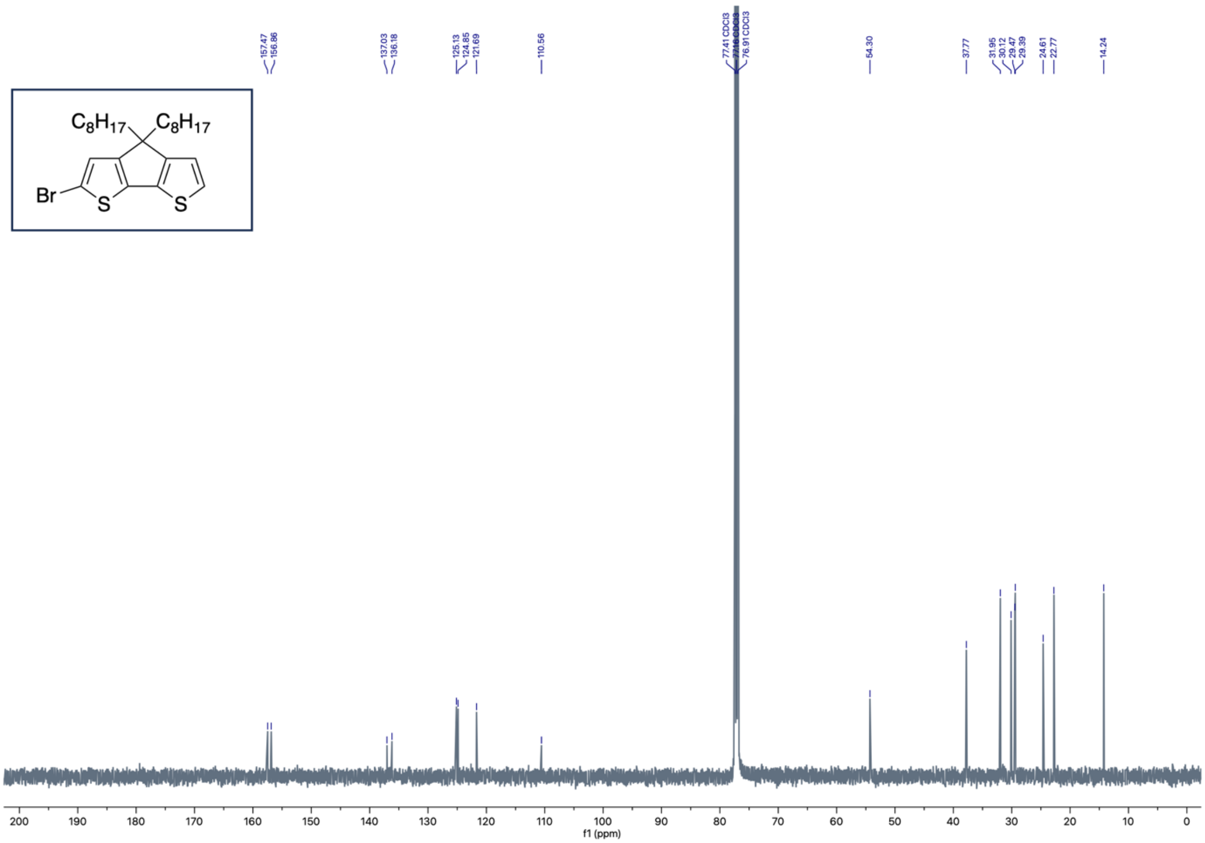
157.47
156.86
137.03
136.18
125.13
124.85
121.69
110.56
77.41 CDCl3
76.91 CDCl3
54.30
37.77
31.95
30.12
29.47
29.39
24.61
22.77
14.24
C8H17
C8H17
Br
S
S
f1 (ppm)

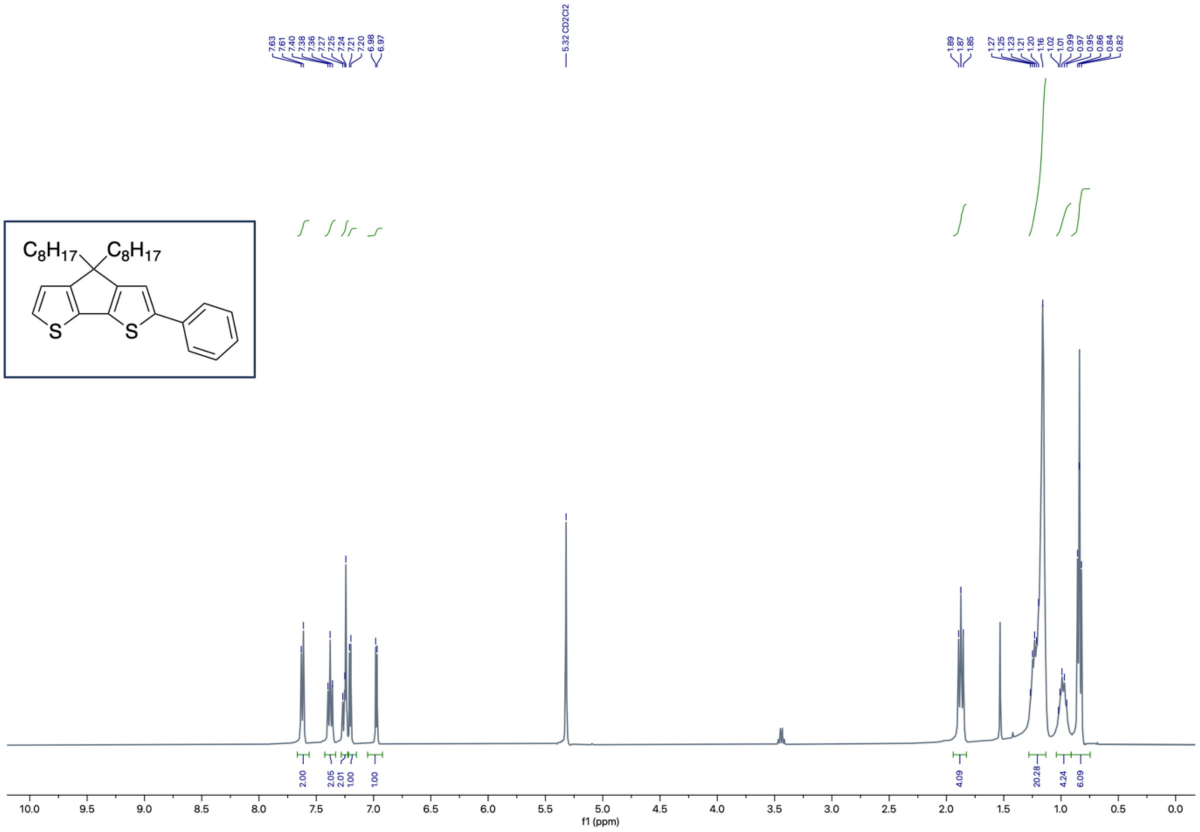

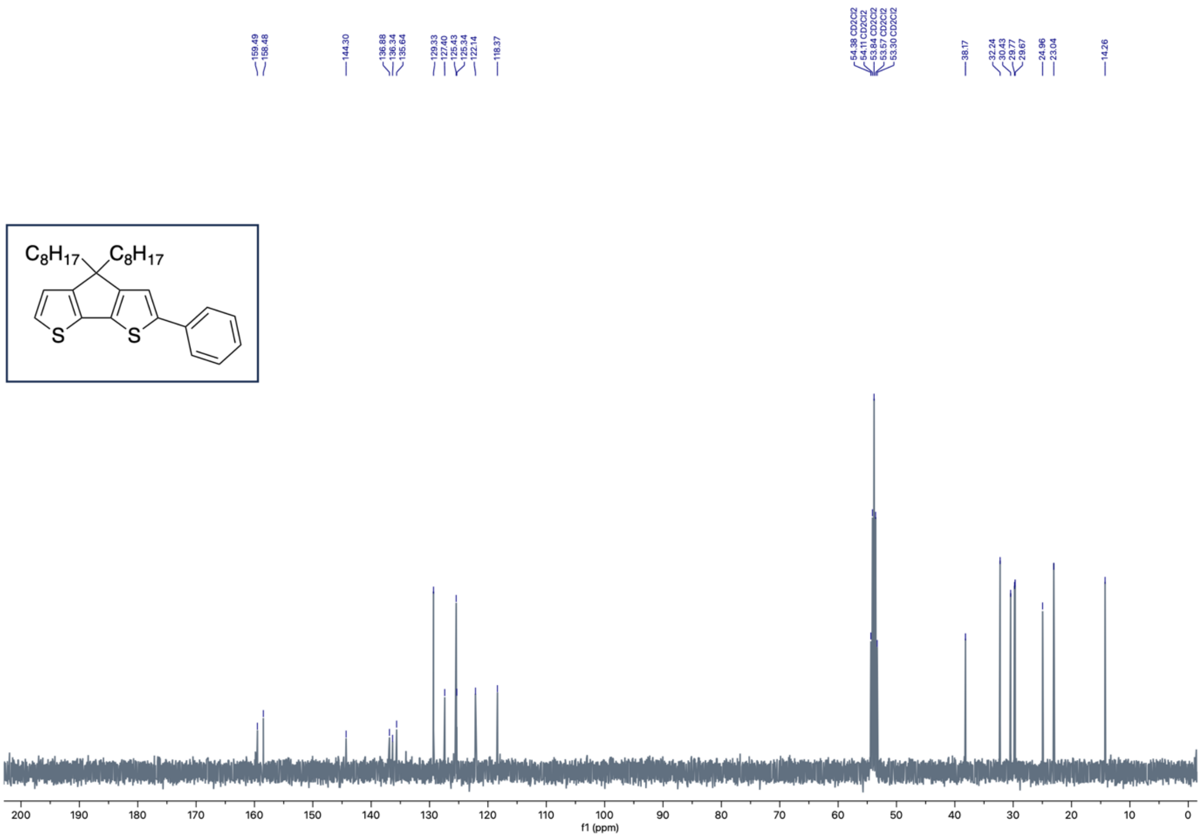
159.49
158.48
144.30
136.88
136.34
135.64
129.33
127.40
125.43
125.34
122.14
118.37
54.38 CD2Cl2
54.11 CD2Cl2
53.84 CD2Cl2
53.57 CD2Cl2
53.30 CD2Cl2
38.17
32.24
30.43
29.77
29.67
24.96
23.04
14.26
C_8H_{17}
C_8H_{17}
S
S
200 190 180 170 160 150 140 130 120 110 100 90 80 70 60 50 40 30 20 10 0
f1 (ppm)

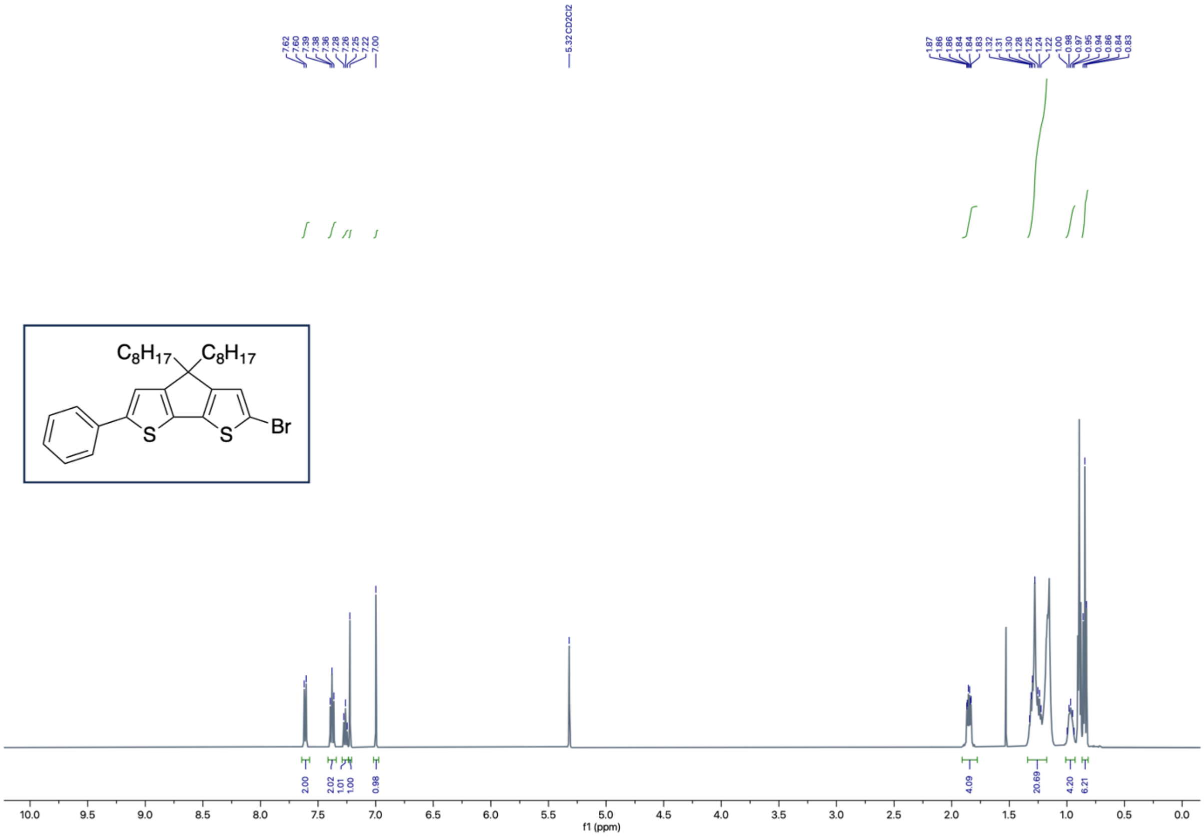

7.62
7.60
7.39
7.38
7.36
7.28
7.26
7.25
7.22
7.00
5.32 CD2Cl2
1.87
1.86
1.86
1.84
1.84
1.83
1.32
1.31
1.30
1.28
1.25
1.24
1.22
1.00
0.98
0.97
0.95
0.94
0.86
0.84
0.83
2.00
2.02
1.01
1.00
0.98
4.09
20.69
4.20
6.21
f1 (ppm)

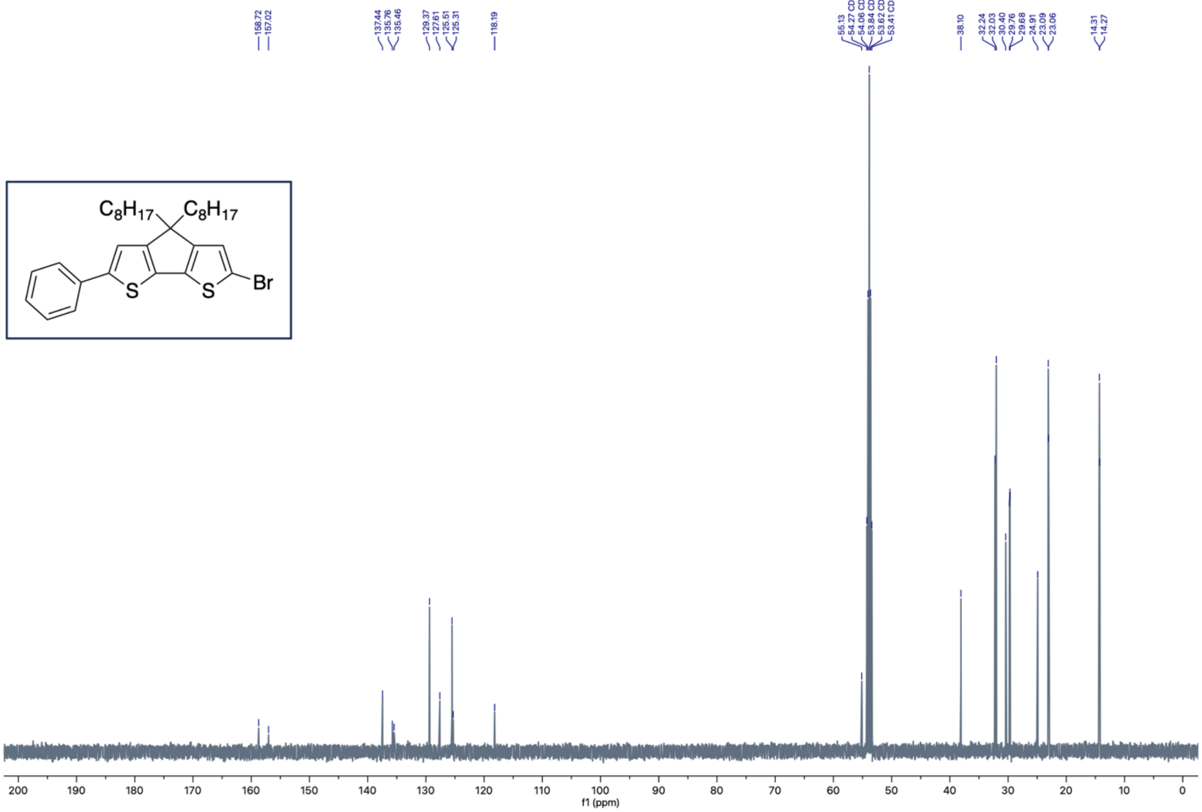

C8H17 C8H17
S S Br
158.72
157.02
137.44
135.76
135.46
129.37
127.61
125.51
125.31
118.19
55.13
54.27 CD2Cl2
54.06 CD2Cl2
53.84 CD2Cl2
53.62 CD2Cl2
53.41 CD2Cl2
38.10
32.24
32.03
30.40
29.76
29.68
24.91
23.09
23.06
14.31
14.27
200 190 180 170 160 150 140 130 120 110 100 90 80 70 60 50 40 30 20 10 0
f1 (ppm)

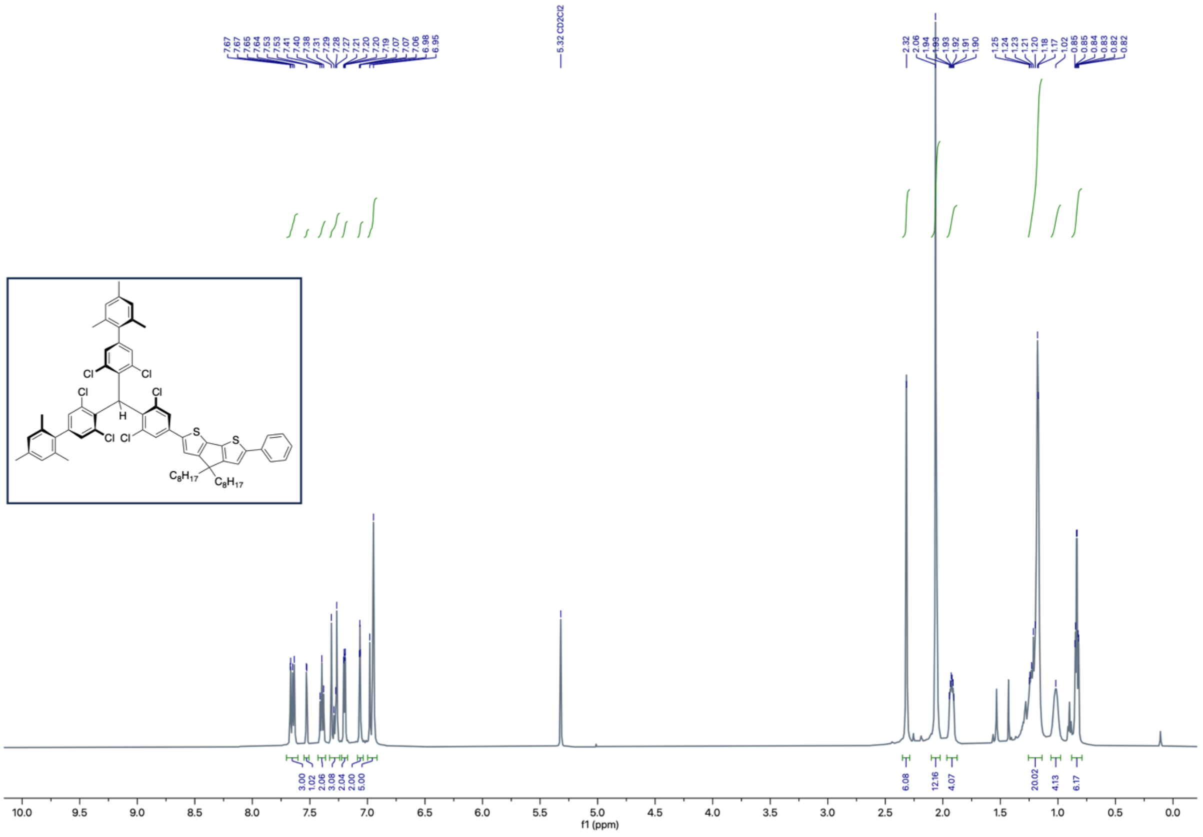

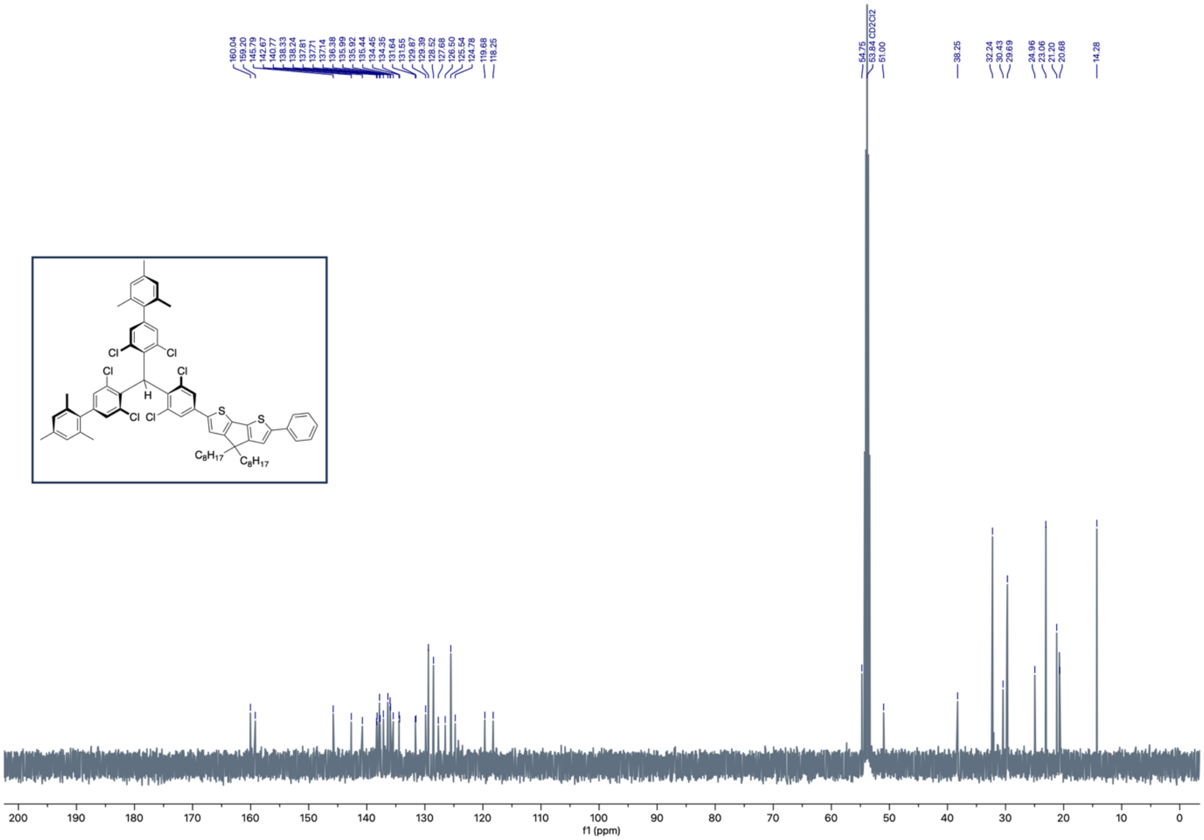

160.04
159.20
145.79
142.67
140.77
138.33
138.24
137.81
137.71
137.14
136.38
135.99
135.92
135.44
134.45
134.35
131.64
131.55
129.87
129.39
128.52
127.68
126.50
125.54
124.78
119.68
118.25
54.75
53.84 CD2Cl2
51.00
38.25
32.24
30.43
29.69
24.96
23.06
21.20
20.68
14.28
Cl
H
S
C_8H_{17}
200 190 180 170 160 150 140 130 120 110 100 90 80 70 60 50 40 30 20 10 0
f1 (ppm)

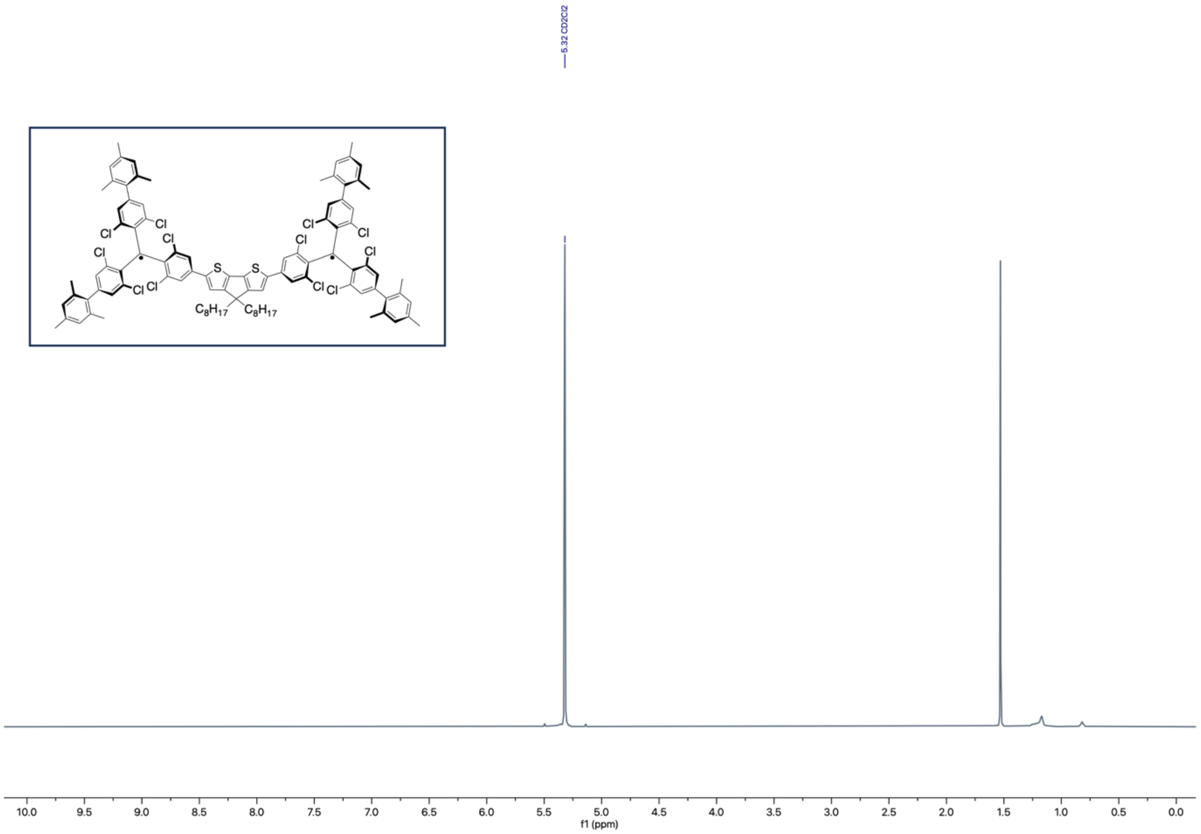

5.32 CD2Cl2
Cl
Cl
S
S
C_8H_{17}
f1 (ppm)

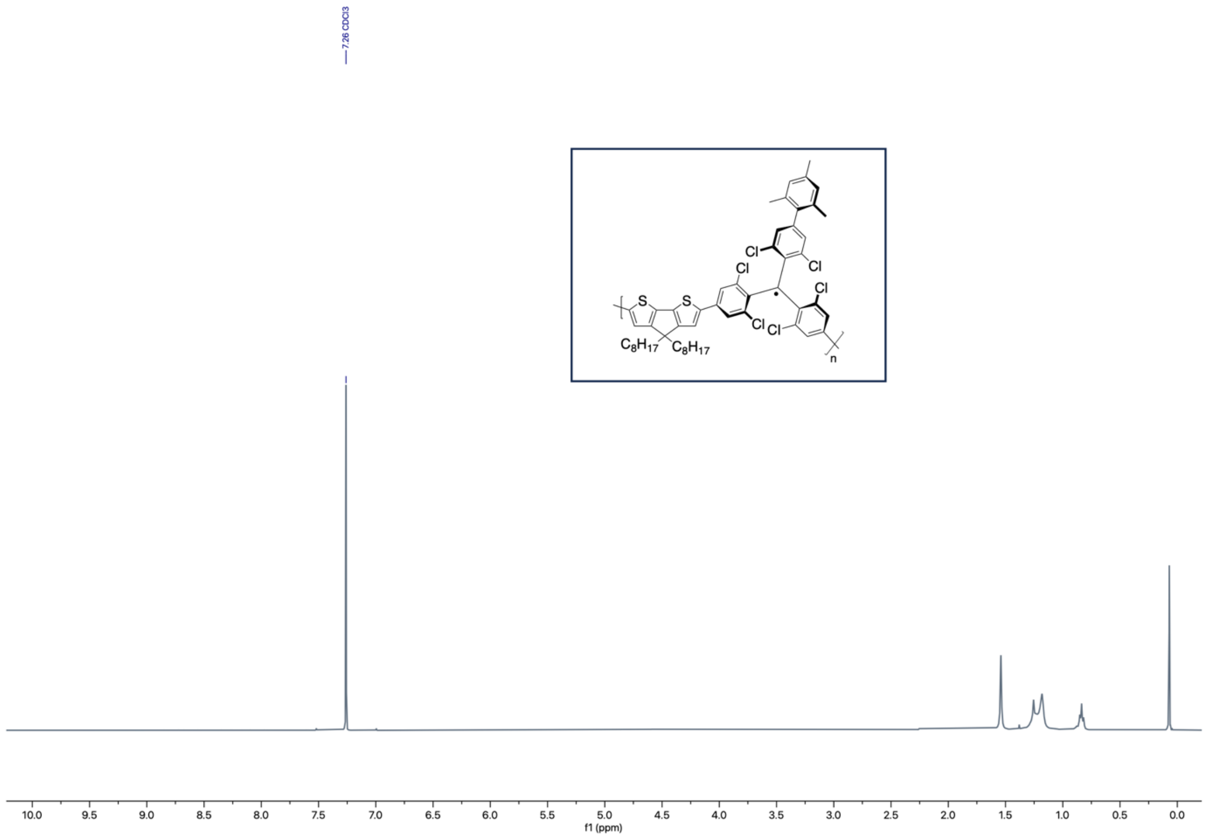
7.26 CDCl3
S
S
Cl
Cl
Cl
Cl
Cl
Cl
C8H17
C8H17
n
10.0
9.5
9.0
8.5
8.0
7.5
7.0
6.5
6.0
5.5
5.0
4.5
4.0
3.5
3.0
2.5
2.0
1.5
1.0
0.5
0.0
f1 (ppm)

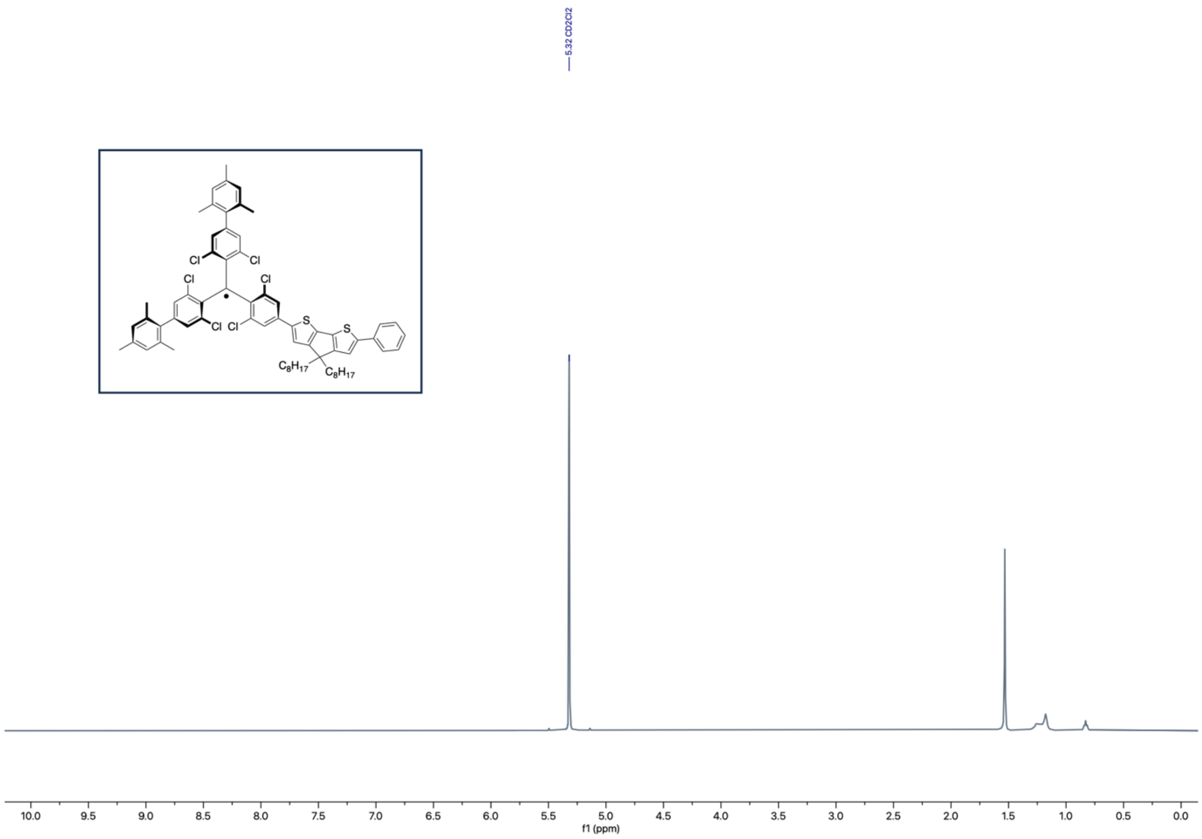

5.32 CD2Cl2
Cl
S
C8H17
10.0
9.5
9.0
8.5
8.0
7.5
7.0
6.5
6.0
5.5
5.0
4.5
4.0
3.5
3.0
2.5
2.0
1.5
1.0
0.5
0.0
f1 (ppm)

REFERENCES AND NOTES

1. X. Ai, E. W. Evans, S. Dong, A. J. Gillett, H. Guo, Y. Chen, T. J. H. Hele, R. H. Friend, F. Li, Efficient radical-based light-emitting diodes with doublet emission. *Nature* **563**, 536–540 (2018).

2. S. A. Wolf, D. D. Awschalom, R. A. Buhrman, J. M. Daughton, S. Von Molnár, M. L. Roukes, A. Y. Chtchelkanova, D. M. Treger, Spintronics: A spin-based electronics vision for the future. *Science* **294**, 1488–1495 (2001).

3. T. P. Nguyen, A. D. Easley, N. Kang, S. Khan, S. M. Lim, Y. H. Rezenom, S. Wang, D. K. Tran, J. Fan, R. A. Letteri, X. He, L. Su, C. H. Yu, J. L. Lutkenhaus, K. L. Wooley, Polypeptide organic radical batteries. *Nature* **593**, 61–66 (2021).

4. Z. X. Chen, Y. Li, F. Huang, Persistent and stable organic radicals: Design, synthesis, and applications. *Chem* **7**, 288–332 (2021).

5. Z. Sun, Q. Ye, C. Chi, J. Wu, Low band gap polycyclic hydrocarbons: From closed-shell near infrared dyes and semiconductors to open-shell radicals. *Chem. Soc. Rev.* **41**, 7857–7889 (2012).

6. L. Ji, J. Shi, J. Wei, T. Yu, W. Huang, L. Ji, J. Shi, J. Wei, T. Yu, W. Huang, Air-stable organic radicals: New-generation materials for flexible electronics? *Adv. Mater.* **32**, e1908015 (2020).

7. G. E. Rudebusch, J. L. Zafra, K. Jorner, K. Fukuda, J. L. Marshall, I. Arrechea-Marcos, G. L. Espejo, R. Ponce Ortiz, C. J. Gómez-García, L. N. Zakharov, M. Nakano, H. Ottosson, J. Casado, M. M. Haley, Diindeno-fusion of an anthracene as a design strategy for stable organic biradicals. *Nat. Chem.* **8**, 753–759 (2016).

8. T. D. Nguyen, E. Ehrenfreund, Z. V. Vardeny, Spin-polarized light-emitting diode based on an organic bipolar spin valve. *Science* **337**, 204–209 (2012).

9. H. Uoyama, K. Goushi, K. Shizu, H. Nomura, C. Adachi, Highly efficient organic light-emitting diodes from delayed fluorescence. *Nature* **492**, 234–238 (2012).

10. P. Murto, H. Bronstein, Electro-optical π-radicals: Design advances, applications and future perspectives. *J. Mater. Chem. C.* **10**, 7368–7403 (2022).

11. L. Zhou, W. Yang, S. Ji, X. Ai, M. T. Sajjad, G. Xie, L. Zhou, W. Yang, S. Ji, X. Ai, M. T. Sajjad, G. Xie, Spin manipulation in organic radicals. *Innov. Mater.* **2**, 100052 (2024).

12. H. Guo, Q. Peng, X. K. Chen, Q. Gu, S. Dong, E. W. Evans, A. J. Gillett, X. Ai, M. Zhang, D. Credgington, V. Coropceanu, R. H. Friend, J. L. Brédas, F. Li, High stability and luminescence efficiency in donor–acceptor neutral radicals not following the Aufbau principle. *Nat. Mater.* **18**, 977–984 (2019).

13. M. Abe, Diradicals. *Chem. Rev.* **113**, 7011–7088 (2013).

14. P. Murto, R. Chowdhury, S. Gorgon, E. Guo, W. Zeng, B. Li, Y. Sun, H. Francis, R. H. Friend, H. Bronstein, Mesitylated trityl radicals, a platform for doublet emission: Symmetry breaking, charge-transfer states and conjugated polymers. *Nat. Commun.* **14**, 4147 (2023).

15. H. U. Kim, T. Kim, C. Kim, M. Kim, T. Park, Recent advances in structural design of efficient near-infrared light-emitting organic small molecules. *Adv. Funct. Mater.* **33**, 2208082 (2023).

16. H. N. Tsao, D. M. Cho, I. Park, M. R. Hansen, A. Mavrinskiy, D. Y. Yoon, R. Graf, W. Pisula, H. W. Spiess, K. Müllen, Ultrahigh mobility in polymer field-effect transistors by design. *J. Am. Chem. Soc.* **133**, 2605–2612 (2011).

17. S. Fan, W. Li, Photonics and thermodynamics concepts in radiative cooling. *Nat. Photonics* **16**, 182–190 (2022).

18. F. Li, A. J. Gillett, Q. Gu, J. Ding, Z. Chen, T. J. H. Hele, W. K. Myers, R. H. Friend, E. W. Evans, Singlet and triplet to doublet energy transfer: Improving organic light-emitting diodes with radicals. *Nat. Commun.* **13**, 2744 (2022).

19. X. Zhu, H. Tsuji, K. Nakabayashi, S.-I. Ohkoshi, E. Nakamura, Air- and Heat-Stable Planar Tri-p-quinodimethane with Distinct Biradical Characteristics. *J. Am. Chem. Soc.* **133**, 16342–16345 (2011).

20. F. Kuriakose, M. Commodore, C. Hu, C. J. Fabiano, D. Sen, R. R. Li, S. Bisht, Ö. Üngör, X. Lin, G. F. Strouse, A. E. Deprince, R. A. Lazenby, F. Mentink-Vigier, M. Shatruk, I. V. Alabugin, Design and

synthesis of Kekulè and non-Kekulè diradicaloids via the radical periannulation strategy: The power of seven Clar’s sextets. *J. Am. Chem. Soc.* **144**, 23448–23464 (2022).

21. X. Lu, S. Lee, J. O. Kim, T. Y. Gopalakrishna, H. Phan, T. S. Herng, Z. Lim, Z. Zeng, J. Ding, D. Kim, J. Wu, Stable 3,6-linked fluorenyl radical oligomers with intramolecular antiferromagnetic coupling and polyradical characters. *J. Am. Chem. Soc.* **138**, 13048–13058 (2016).

22. X. Lu, S. Lee, Y. Hong, H. Phan, T. Y. Gopalakrishna, T. S. Herng, T. Tanaka, M. E. Sandoval-Salinas, W. Zeng, J. Ding, D. Casanova, A. Osuka, D. Kim, J. Wu, Fluorenyl based macrocyclic polyradicaloids. *J. Am. Chem. Soc.* **139**, 13173–13183 (2017).

23. M. S. Vezie, S. Few, I. Meager, G. Pieridou, B. Dörling, R. S. Ashraf, A. R. Goñi, H. Bronstein, I. McCulloch, S. C. Hayes, M. Campoy-Quiles, J. Nelson, Exploring the origin of high optical absorption in conjugated polymers. *Nat. Mater.* **15**, 746–753 (2016).

24. Q. Peng, A. Blikim Obolda, M. Zhang, F. Li, Organic light-emitting diodes using a neutral π radical as emitter: The emission from a doublet. *Angew. Chem. Int. Ed.* **127**, 7197–7201 (2015).

25. A. Abdurahman, Q. Peng, O. Ablikim, X. Ai, F. Li, A radical polymer with efficient deep-red luminescence in the condensed state. *Mater. Horiz.* **6**, 1265–1270 (2019).

26. A. Abdurahman, T. J. H. Hele, Q. Gu, J. Zhang, Q. Peng, M. Zhang, R. H. Friend, F. Li, E. W. Evans, Understanding the luminescent nature of organic radicals for efficient doublet emitters and pure-red light-emitting diodes. *Nat. Mater.* **19**, 1224–1229 (2020).

27. Z. Cui, A. Abdurahman, X. Ai, F. Li, Stable luminescent radicals and radical-based LEDs with doublet emission. *CCS Chem.* **2**, 1129–1145 (2020)

28. Z. Sun, K. W. Huang, J. Wu, Soluble and stable heptazethrenebis(dicarboximide) with a singlet open-shell ground state. *J. Am. Chem. Soc.* **133**, 11896–11899 (2011).

29. S. Das, T. S. Herng, J. L. Zafra, P. M. Burrezo, M. Kitano, M. Ishida, T. Y. Gopalakrishna, P. Hu, A. Osuka, J. Casado, J. Ding, D. Casanova, J. Wu, Fully fused quinoidal/aromatic carbazole macrocycles with poly-radical characters. *J. Am. Chem. Soc.* **138**, 7782–7790 (2016).

30. J. J. Dressler, M. Teraoka, G. L. Espejo, R. Kishi, S. Takamuku, C. J. Gómez-García, L. N. Zakharov, M. Nakano, J. Casado, M. M. Haley, Thiophene and its sulfur inhibit indenoindenodibenzothiophene diradicals from low-energy lying thermal triplets. *Nat. Chem.* **10**, 1134–1140 (2018).

31. X. Song, X. Lu, B. Sun, H. Zhang, P. Sun, H. Miao, Q. Fan, W. Huang, Conjugated polymer nanoparticles with absorption beyond 1000 nm for NIR-II fluorescence imaging system guided NIR-II photothermal therapy. *ACS Appl. Polym. Mater.* **2**, 4171–4179 (2020).

32. M. Nakamura, M. Gon, K. Tanaka, Y. Chujo, Solid-state near-infrared emission of π-conjugated polymers consisting of boron complexes with vertically projected steric substituents. *Macromolecules* **56**, 2709–2718 (2023).

33. M. Nakamura, I. Kanetani, M. Gon, K. Tanaka, NIR-II absorption/fluorescence of D–A π-conjugated polymers composed of strong electron acceptors based on boron-fused azobenzene complexes. *Angew. Chem. Int. Edition*, **63**, e202404178 (2024).

34. K. Andersson, P.-Á. Malmqvist, B. O. Roos, A. J. Sadlej, K. Wolinski, Second-order perturbation theory with a CASSCF reference function. *J. Phys. Chem.* **94**, 5483–5488 (1990).

35. L. Salem, C. Rowland, The electronic properties of diradicals. *Angew. Chem. Int. Ed.* **11**, 92–111 (1972).

36. T. Stuyver, B. Chen, T. Zeng, P. Geerlings, F. De Proft, R. Hoffmann, Do diradicals behave like radicals? *Chem. Rev.* **119**, 11291–11351 (2019).

37. P.Kimber, F.Plasser, "*Classification and Analysis of Molecular Excited States. In Comprehensive Computational Chemistry*". (Elsevier, 2024), pp. 55–83.

38. D. S. Engebretson, J. M. Zaleski, G. E. Leroi, D. G. Nocera, Direct spectroscopic detection of a zwitterionic excited state. *Science* **265**, 759–762 (1994).

39. F. Miao, Y. Ji, B. Han, S. M. Quintero, H. Chen, G. Xue, L. Cai, J. Casado, Y. Zheng, Asymmetric and zwitterionic Blatter diradicals. *Chem. Sci.* **14**, 2698–2705 (2023).

40. C. H. Liu, Z. He, C. Ruchlin, Y. Che, K. Somers, D. F. Perepichka, Thiele's fluorocarbons: Stable diradicaloids with efficient visible-to-near-infrared fluorescence from a zwitterionic excited state. *J. Am. Chem. Soc.* **145**, 15702–15707 (2023).

41. W. L. Jiang, Z. Peng, B. Huang, X. L. Zhao, D. Sun, X. Shi, H. B. Yang, TEMPO radical-functionalized supramolecular coordination complexes with controllable spin-spin interactions. *J. Am. Chem. Soc.* **143**, 433–441 (2021).

42. W. B. Gleason, R. E. Barnett, Use of the point dipole approximation for nitroxide biradicals. *J. Am. Chem. Soc.* **98**, 2701–2705 (1976).

43. C. E. Tait, P. Neuhaus, M. D. Peeks, H. L. Anderson, C. R. Timmel, Transient EPR reveals triplet state delocalization in a series of cyclic and linear π-conjugated porphyrin oligomers. *J. Am. Chem. Soc.* **137**, 8284–8293 (2015).

44. C. E. Tait, P. Neuhaus, H. L. Anderson, C. R. Timmel, Triplet state delocalization in a conjugated porphyrin dimer probed by transient electron paramagnetic resonance techniques. *J. Am. Chem. Soc.* **137**, 6670–6679 (2015).

45. A. Abdurahman, J. Wang, Y. Zhao, P. Li, L. Shen, Q. Peng, A highly stable organic luminescent diradical. *Angew. Chem. Int. Ed.* **62**, e202300772 (2023).

46. G. Chiappe, E. Louis, A. Guijarro, E. San-Fabián, J. A. Vergés, Exponential decay of spin-spin correlation between distant defect states produced by contour hydrogenation of polycyclic aromatic hydrocarbon molecules. *Phys. Rev. B* **87**, 125126 (2013).

47. J. Richter, D. Schubert, R. Steinigeweg, Decay of spin-spin correlations in disordered quantum and classical spin chains. *Phys. Rev. Res.* **2**, 013130 (2020).

48. S. Mugiraneza, A. M. Hallas, Tutorial: A beginner's guide to interpreting magnetic susceptibility data with the Curie-Weiss law. *Commun. Phys.* **5**, 1–12 (2022).

49. T. Jungwirth, X. Marti, P. Wadley, J. Wunderlich, Antiferromagnetic spintronics. *Nat. Nanotechnol.* **11**, 231–241 (2016).

50. E. Rongione, O. Gueckstock, M. Mattern, O. Gomonay, H. Meer, C. Schmitt, R. Ramos, T. Kikkawa, M. Mičica, E. Saitoh, J. Sinova, H. Jaffrès, J. Mangeney, S. T. B. Goennenwein, S. Geprägs, T. Kampfrath, M. Kläui, M. Bargheer, T. S. Seifert, S. Dhillon, R. Lebrun, Emission of coherent THz magnons in an antiferromagnetic insulator triggered by ultrafast spin–phonon interactions. *Nat. Commun.* **14**, 1818 (2023).

51. M. Souto, V. Lloveras, S. Vela, M. Fumanal, I. Ratera, J. Veciana, Three redox states of a diradical acceptor-donor-acceptor triad: Gating the magnetic coupling and the electron delocalization. *J. Phys. Chem. Lett.* **7**, 2234–2239 (2016).

52. Y. Yamashita, J. Tsurumi, M. Ohno, R. Fujimoto, S. Kumagai, T. Kurosawa, T. Okamoto, J. Takeya, S. Watanabe, Efficient molecular doping of polymeric semiconductors driven by anion exchange. *Nature* **572**, 634–638 (2019).

53. F. Yu, W. Liu, S. W. Ke, M. Kurmoo, J. L. Zuo, Q. Zhang, Electrochromic two-dimensional covalent organic framework with a reversible dark-to-transparent switch. *Nat. Commun* **11**, 5534 (2020).

54. R. M. Pankow, A. Harbuzaru, D. Zheng, B. Kerwin, G. Forti, I. D. Duplessis, B. Musolino, R. Ponce Ortiz, A. Facchetti, T. J. Marks, Oxidative-reductive near-infrared electrochromic switching enabled by porous vertically stacked multilayer devices. *J. Am. Chem. Soc.* **145**, 13411–13419 (2023).

55. K. Pierloot, B. Dumez, P. O. Widmark, B. O. Roos, Density matrix averaged atomic natural orbital (ANO) basis sets for correlated molecular wave functions. *Theor. Chim. Acta* **90**, 87–114 (1995).

56. G. Li Manni, I. F. Galván, A. Alavi, F. Aleotti, F. Aquilante, J. Autschbach, D. Avagliano, A. Baiardi, J. J. Bao, S. Battaglia, L. Birnoschi, A. Blanco-González, S. I. Bokarev, R. Broer, R. Cacciari, P. B. Calio, R. K. Carlson, R. Carvalho Couto, L. Cerdán, L. F. Chibotaru, N. F. Chilton, J. R. Church, I. Conti, S. Coriani, J. Cuéllar-Zuquin, R. E. Daoud, N. Dattani, P. Decleva, C. de Graaf, M. G. Delcey, L. De Vico, W. Dobrautz, S. S. Dong, R. Feng, N. Ferré, M. Filatov, L. Gagliardi, M. Garavelli, L. González, Y. Guan, M. Guo, M. R. Hennefarth, M. R. Hermes, C. E. Hoyer, M. Huix-Rotllant, V. K. Jaiswal, A. Kaiser, D. S. Kaliakin, M. Khamesian, D. S. King, V. Kochetov, M. Krośnicki, A. A. Kumaar, E. D. Larsson, S. Lehtola, M. B. Lepetit, H. Lischka, P. López Ríos, M. Lundberg, D. Ma, S. Mai, P. Marquetand, I. C. D. Merritt, F. Montorsi, M. Mörchen, A. Nenov, V. H. A. Nguyen, Y.

Nishimoto, M. S. Oakley, M. Olivucci, M. Oppel, D. Padula, R. Pandharkar, Q. M. Phung, F. Plasser, G. Raggi, E. Rebolini, M. Reiher, I. Rivalta, D. Roca-Sanjuán, T. Romig, A. A. Safari, A. Sánchez-Mansilla, A. M. Sand, I. Schapiro, T. R. Scott, J. Segarra-Martí, F. Segatta, D. C. Sergentu, P. Sharma, R. Shepard, Y. Shu, J. K. Staab, T. P. Straatsma, L. K. Sørensen, B. N. C. Tenorio, D. G. Truhlar, L. Ungur, M. Vacher, V. Veryazov, T. A. Voß, O. Weser, D. Wu, X. Yang, D. Yarkony, C. Zhou, J. P. Zobel, R. Lindh, The OpenMolcas Web: A community-driven approach to advancing computational chemistry. *J. Chem. Theory Comput.* **19**, 6933–6991 (2023).

57. F. Neese, Software update: The ORCA program system—Version 5.0. *Wiley Interdiscip. Rev. Comput. Mol. Sci.* **12**, e1606 (2022).

58. S. Stoll, A. Schweiger, EasySpin, a comprehensive software package for spectral simulation and analysis in EPR. *J. Magn. Reson.* **178**, 42–55 (2006).

59. M. J. Frisch, G. W. Trucks, H. B. Schlegel, G. E. Scuseria, M. A. Robb, J. R. Cheeseman, G. Scalmani, V. Barone, G. A. Petersson, H. Nakatsuji, X. Li, M. Caricato, A. V. Marenich, J. Bloino, B. G. Janesko, R. Gomperts, B. Mennucci, H. P. Hratchian, J. V. Ortiz, A. F. Izmaylov, J. L. Sonnenberg, D. Williams-Young, F. Ding, F. Lipparini, F. Egidi, J. Goings, B. Peng, A. Petrone, T. Henderson, D. Ranasinghe, V. G. Zakrzewski, J. Gao, N. Rega, G. Zheng, W. Liang, M. Hada, M. Ehara, K. Toyota, R. Fukuda, J. Hasegawa, M. Ishida, T. Nakajima, Y. Honda, O. Kitao, H. Nakai, T. Vreven, K. Throssell, J. A. Montgomery, Jr., J. E. Peralta, F. Ogliaro, M. J. Bearpark, J. J. Heyd, E. N. Brothers, K. N. Kudin, V. N. Staroverov, T. A. Keith, R. Kobayashi, J. Normand, K. Raghavachari, A. P. Rendell, J. C. Burant, S. S. Iyengar, J. Tomasi, M. Cossi, J. M. Millam, M. Klene, C. Adamo, R. Cammi, J. W. Ochterski, R. L. Martin, K. Morokuma, O. Farkas, J. B. Foresman, D. J. Fox, Gaussian 16, Rev. C.01. *Gaussian 16, Rev. C. 01* (2016).

60. C. Hwang, J. Lee, J. Jeong, E. Lee, J. Kim, S. Kim, C. Yang, H. K. Song, The rational design of a redox-active mixed ion/electron conductor as a multi-functional binder for lithium-ion batteries. *J. Mater. Chem. A* **9**, 4751–4757 (2021).

61. L. Yuan, S. Liang, C. Xiao, Q. Chen, W. Li, Near-infrared nonfullerene acceptors based on 4H-cyclopenta[1,2-b:5,4-b′]dithiophene for organic solar cells and organic field-effect transistors. *Chem. Asian J.* **16**, 4171–4178 (2021).

62. F. Plasser, A. I. Krylov, A. Dreuw, libwfa: Wavefunction analysis tools for excited and open-shell electronic states. *Wiley Interdiscip. Rev. Comput. Mol. Sci.* **12**, e1595 (2022).
